# Comparative transcriptome analysis reveals cadmium tolerance mechanisms in two *Amaranthus* varieties

**DOI:** 10.3389/fpls.2026.1724716

**Published:** 2026-02-12

**Authors:** Taotao Wang, Fuqiang Zhu, Ying Liu, Ningning Xing, Yuan Chen

**Affiliations:** State Key Laboratory of Wheat Improvement, Peking University Institute of Advanced Agricultural Sciences, Shandong Laboratory of Advanced Agricultural Sciences in Weifang, Weifang, Shandong, China

**Keywords:** *Amaranthus*, cadmium tolerance, differentially expressed genes (DEGs), heavy metal transport, transcription factors, transcriptome analysis

## Abstract

Cadmium (Cd) pollution poses a significant threat to agricultural safety and ecological health. Understanding plant tolerance mechanisms is crucial for developing solutions. In this study, we investigated the physiological and molecular mechanisms underlying Cd tolerance in two *Amaranthus* varieties, CZ074 (tolerant) and CZ081 (sensitive). Physiological analysis revealed CZ074 exhibited less severe growth inhibition under Cd stress. Notably, CZ074 exhibited a lower Cd transport coefficient from roots to shoots, which contributes to protecting its aboveground tissues from toxicity. Comparative transcriptome analysis of roots, stems, and leaves identified a significantly larger number of differentially expressed genes (DEGs) in CZ074, suggesting an “active investment” strategy involving comprehensive transcriptional reprogramming. In CZ074 roots, DEGs were markedly enriched in pathways related to phenylpropanoid biosynthesis, cell wall formation (cutin, suberine, and wax), ribosome biogenesis, and plant hormone signal transduction. Key transcription factors (*bZIP*, *NAC*, *WRKY*) and transporter genes (*HMA3*, *NRAMPs*, *CAXs*) were more strongly up-regulated in CZ074 roots, indicating enhanced defense, sequestration, and transport capabilities. In shoots, CZ081 showed more dysregulated gene expression, interpreted as a symptom of severe stress damage rather than an effective defense. We propose that CZ074’s tolerance stems from a coordinated network involving robust root defenses, efficient Cd translocation in stems, all supported by extensive and precise transcriptional regulation. These findings provide valuable candidate genes and insights for phytoremediation and breeding Cd-tolerant crops.

## Introduction

With the rapid development of industry and agriculture, soil heavy metal pollution has become a global environmental issue (Yang et al., 2018). Cadmium (Cd), due to its high toxicity, strong mobility, and propensity to accumulate in the food chain, poses a serious threat to agricultural product safety and human health (Rani et al., 2014; Genchi et al., 2020). Under Cd stress, plants suffer from a series of physiological damages, including oxidative damage, nutritional imbalance, inhibition of photosynthesis, and impaired growth and development (Aslam et al., 2023; Mushtaq et al., 2025). Therefore, elucidating the mechanisms of Cd tolerance in plants is of significant theoretical and practical importance for cultivating low-Cd-accumulating crops or using plants for the phytoremediation of Cd-contaminated soils.

Over the course of long-term evolution, plants have developed a complex set of mechanisms to cope with Cd stress, including root cell wall binding, vacuolar sequestration, chelation, and antioxidant defense, involving numerous regulatory factors such as zinc/iron-regulated transporter proteins (ZIP), natural resistance-associated macrophage proteins (Nramp) transporters, and heavy metal ATPase (HMA) transporters (Vitelli et al., 2024; Zhang et al., 2024b; Hu et al., 2025). Underlying these physiological processes is a precise transcriptional regulatory network. In recent years, transcriptomic technologies (e.g., RNA-seq) have become powerful tools for genome-wide revelation of plant responses to abiotic stresses, enabling the systematic identification of key differentially expressed genes and regulatory pathways (Wang et al., 2024). However, Cd tolerance strategies vary significantly among different plant species and even genotypes, and this genetic diversity is key to mining excellent tolerance gene resources (He et al., 2015; Yang et al., 2025).

*Amaranthus*, a multi-purpose plant with value for food, feed, and ornamental uses, exhibits strong environmental adaptability and a certain potential for heavy metal tolerance (Hunková et al., 2024; Mukuwapasi et al., 2024). Preliminary studies have found significant differences in Cd stress tolerance among different amaranth varieties, including *A hybridus, A. cruentus, A. mangostanus, A. tricolor, A. retroflexus, A.* sp*inosus, A. dubius*, and *A. panuiculatus*, making them ideal materials for comparative study of Cd tolerance mechanisms at the molecular level (Lancíková et al., 2020; Hunková et al., 2024). However, research on Cd tolerance in amaranth, particularly systematic comparisons of regulatory mechanisms between varieties with different tolerance levels at the whole transcriptome level, is still lacking.

In this study, we selected two *Amaranthus* varieties with contrasting Cd tolerance: tolerant CZ074 (*A. cruentus*) and sensitive CZ081 (*A. hybridus*), to investigate their phenotypic changes and Cd accumulation characteristics CdCl_2_ treatment. Using RNA-seq, we further compared their transcriptional responses in root, stem, and leaves (**Supplementary Figure 1**). The specific objectives were to: (1) clarify the physiological and Cd accumulation differences between the two varieties; (2) reveal their global gene expression profile changes under Cd stress; (3) identify key genes, biological pathways, and transcriptional regulatory networks associated with Cd tolerance. This study aims to This study aims to elucidate the transcriptional mechanisms underlying Cd tolerance in amaranth, thereby offering both theoretical insights and gene resources for improving crop Cd resistance and for selecting plant materials suitable for phytoremediation.

## Materials and methods

### Plant material growth and treatment conditions

Seeds of amaranth (varieties CZ074 and CZ081 were obtained from the Chinese Academy of Agricultural Sciences (from Guangxi and Guizhou provinces, respectively). The seeds were germinated in a vermiculite substrate for 7 days and then transferred to a hydroponic system containing half-strength Hoagland nutrient solution. They were cultivated until the 4th-5th true leaf stage before initiating Cd treatment (Haider et al., 2021). For the treatment, CdCl_2_ was added to the half-strength Hoagland solution at four concentration gradients: 0 mg/L (control), 1 mg/L, 2 mg/L, and 3 mg/L. The nutrient solution was replaced every 5 days to maintain a stable growth environment. After 10 days of treatment, plant height, SPAD, aboveground and root fresh weight were measured and analyzed using the two-way ANOVA (Tukey’s *post hoc* test). The Cd transport coefficient of Cd was calculated as the ratio of the Cd concentration in the aboveground tissues to that in the roots (Liu et al., 2013).

### Detection of Cd content in plant tissues

Root, stem, and leaf tissue samples from CZ074 and CZ081 plants treated with 1 mg/L Cd were collected separately. After rinsing with deionized water, the samples were dried in an oven at 80 °C until a constant weight was achieved. The dried tissues were ground and sieved. The samples were then subjected to a pre-treatment digestion process using concentrated nitric acid (HNO_3_). The Cd concentration in the digestate was determined using Inductively Coupled Plasma Mass Spectrometry (ICP-MS) to analyze the Cd accumulation capacity in different tissues.

Cd dynamic translocation analysis was conducted: (1) root, stem, and leaf samples of CZ074 and CZ081 were collected at 0, 12, 24, and 72 h after 1 mg/L Cd treatment (three biological replicates per time point) and assayed according to the aforementioned protocol; (2) After 72 h treatment, xylem sap was collected from stems via vacuum extraction (0.05–0.08 MPa for 30 min), filtered through a 0.22 μm nylon filter, and directly analyzed by ICP-MS to quantify Cd long-distance transport efficiency. Blank controls were included, and ICP-MS was calibrated with 0.01–100 μg/L Cd standard solutions for accuracy.

### Isolation of total RNA, cDNA library construction, and illumina sequencing

Root, stem, and leaf tissues from CZ074 and CZ081 plants treated with 0 and 1 mg/L Cd were collected separately. Three biological replicates were taken for each treatment and immediately flash-frozen in liquid nitrogen. Total RNA was extracted from the different amaranth samples using the Trizol method. The integrity and quantity of the RNA were accurately assessed using an Agilent 2100 Bioanalyzer (Agilent Technologies, CA, USA). RNA samples meeting the quality requirements were sent for sequencing (Novogene Bioinformatics Technology Co., Ltd., Tianjin, China). Subsequently, cDNA libraries were constructed using the NEB standard or strand-specific methods. The constructed libraries were quantified using Qubit and quality-checked using a Bioanalyzer. Sequencing was performed on the Illumina NovaSeq 6000 platform using the PE150 (Paired-End 150 bp) strategy and Sequencing by Synthesis (SBS) technology, generating at least 6 Gb of clean data per sample. The original data can be downloaded from NCBI (PRJNA1345021).

### Transcriptome assembly and gene annotation

The raw sequencing data were processed using CASAVA for base calling and filtered to obtain clean reads. *De novo* transcriptome assembly was performed using Trinity (version v2.5.1) software, specifically designed for transcriptome assembly. The assembly process involved: the Inchworm module assembling contigs from clean reads; the Chrysalis module constructing de Bruijn graphs from the contigs; and the Butterfly module resolving alternative splicing and outputting full-length transcripts. Redundant transcripts were clustered into “Genes” using Corset (https://code.google.com/p/corset-project/). The assembly quality of the resulting clusters and unigenes was evaluated using BUSCO (Benchmarking Universal Single-Copy Orthologs; http://busco.ezlab.org/), assessing the percentage and completeness of matched universal single-copy orthologs to evaluate the accuracy and completeness of the assembly. All assembled amaranth genes were functionally annotated against databases including GO (Gene Ontology), KEGG (Kyoto Encyclopedia of Genes and Genomes), Nr (NCBI non-redundant protein sequences), Nt (NCBI nucleotide sequences), Pfam (Protein family), KOG/COG (Clusters of Orthologous Groups), and Swiss-Prot (a manually annotated and reviewed protein sequence database).

### Transcriptome data analysis

Raw reads were processed to remove adapters, poly-N sequences, and low-quality reads. Quality metrics, including Q20, Q30, and GC content, were calculated for the clean data. All subsequent analyses were performed using this high-quality processed dataset. The transcriptome assembled by Trinity was used as the reference sequence. Clean reads from each sample were mapped to this reference transcriptome using HISAT2 (v2.0.5). The read count for each gene was then obtained using RSEM software based on the alignment results. Differential expression analysis between comparison groups was performed using the DESeq2 software (version 1.20.0). Genes with an absolute value of log_2_ fold change greater than or equal to 1 and an adjusted p-value (padj) < 0.05 were identified as differentially expressed.

### Enrichment analysis of differentially expressed genes

Functional enrichment analysis of the differentially expressed gene (DEG) sets, including GO term enrichment and KEGG pathway enrichment, was performed using the clusterProfiler software. This analysis is based on the hypergeometric distribution principle. The “test gene set” consisted of the significantly differentially expressed genes that were successfully annotated in the GO or KEGG databases. The “background gene set” consisted of all genes included in the differential expression analysis that were annotated in the respective GO or KEGG databases. Enrichment analysis was conducted for all DEG sets, up-regulated DEG sets, and down-regulated DEG sets from each differential comparison combination.

### Data statistics and analysis

If not otherwise specified, statistical differences were calculated using Student’s t-test (unpaired, two-tailed) in SPSS software. Significance levels were defined as *P < 0.05, **P < 0.01, ***P < 0.001, and ****P < 0.0001. Bar charts were generated using GraphPad Prism 8. Heatmaps were generated, and sequence alignments were performed using TBtools (https://github.com/CJ-Chen/TBtools-II). All figures were assembled and finalized using Adobe Illustrator CS6.

## Results

### CZ074 exhibited stronger Cd stress tolerance than CZ081

**Figure 1:**
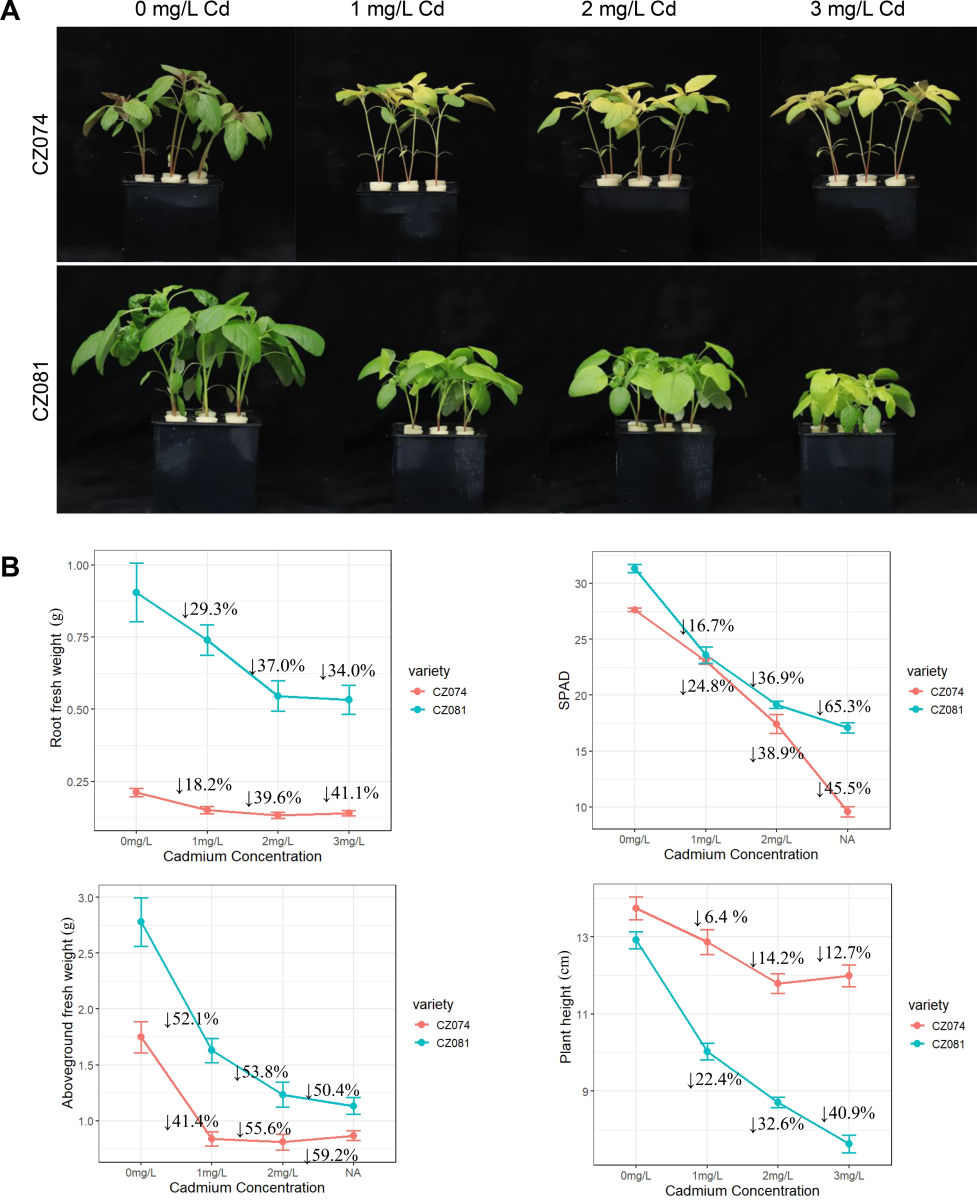
Effects of cadmium chloride treatment on phenotype and growth of amaranth. **(A)** Phenotype of two amaranth varieties (CZ074 and CZ081) under different concentrations of CdCl_2_ treatment (0, 1, 2, 3 mg/L). Bar = 5 cm; **(B)** Line chart of plant height, fresh weight, and chlorophyll content of the two varieties under different CdCl_2_ concentrations. Data are presented as mean ± SD (n ≥ 15).

To evaluate the Cd stress tolerance of the two amaranth varieties (CZ074 and CZ081), they were treated with different concentrations (0, 1, 2, 3 mg/L) of CdCl_2_. The treatment results showed that with increasing CdCl_2_ concentration, both varieties exhibited typical Cd stress symptoms (**Supplementary Table 1**). Compared to the control group (0 mg/L), the treated plants showed significant growth inhibition, specifically manifested as leaf chlorosis, reduced biomass, and plant dwarfing (**Figure 1A**).

We quantified the decline rates of aboveground fresh weight, root fresh weight, chlorophyll content, and plant height in response to Cd treatment. Cd stress significantly inhibited the growth of both cultivars in a dose-dependent manner (**Figure 1B**). Compared with the control (0 mg/L), root fresh weight decreased by 33.3% in CZ074 and 41.8% in CZ081, while aboveground fresh weight declined by 50.3% in CZ074 and 59.2% in CZ081 at 3 mg/L Cd. In addition, plant height was reduced by 12.7% in CZ074, but by 40.9% in CZ081 under the same treatment.

Although CZ081 exhibited relatively better chlorophyll retention and slight advantages in aboveground and root growth under low Cd conditions, biomass-related parameters are generally considered more robust indicators of Cd stress sensitivity, as reported previously (Guo et al., 2019; García De La Torre et al., 2021; Yildirim et al., 2023). Based on this core biomass assessment, CZ074 displayed greater growth stability under intensified Cd stress. Notably, significant varietal differences were already evident at 1 mg/L Cd, and this concentration was therefore selected for subsequent experiments.

### Amaranth transcriptome sequencing, assembly, and functional annotation

Due to the lack of a high-quality reference genome for amaranth, we performed RNA-seq analysis on the roots, stems, and leaves of the two varieties (CZ074 and CZ081) under treatment with 1 mg/L CdCl_2_, and constructed the transcriptome using a *de novo* assembly strategy in this study.

**Figure 2:**
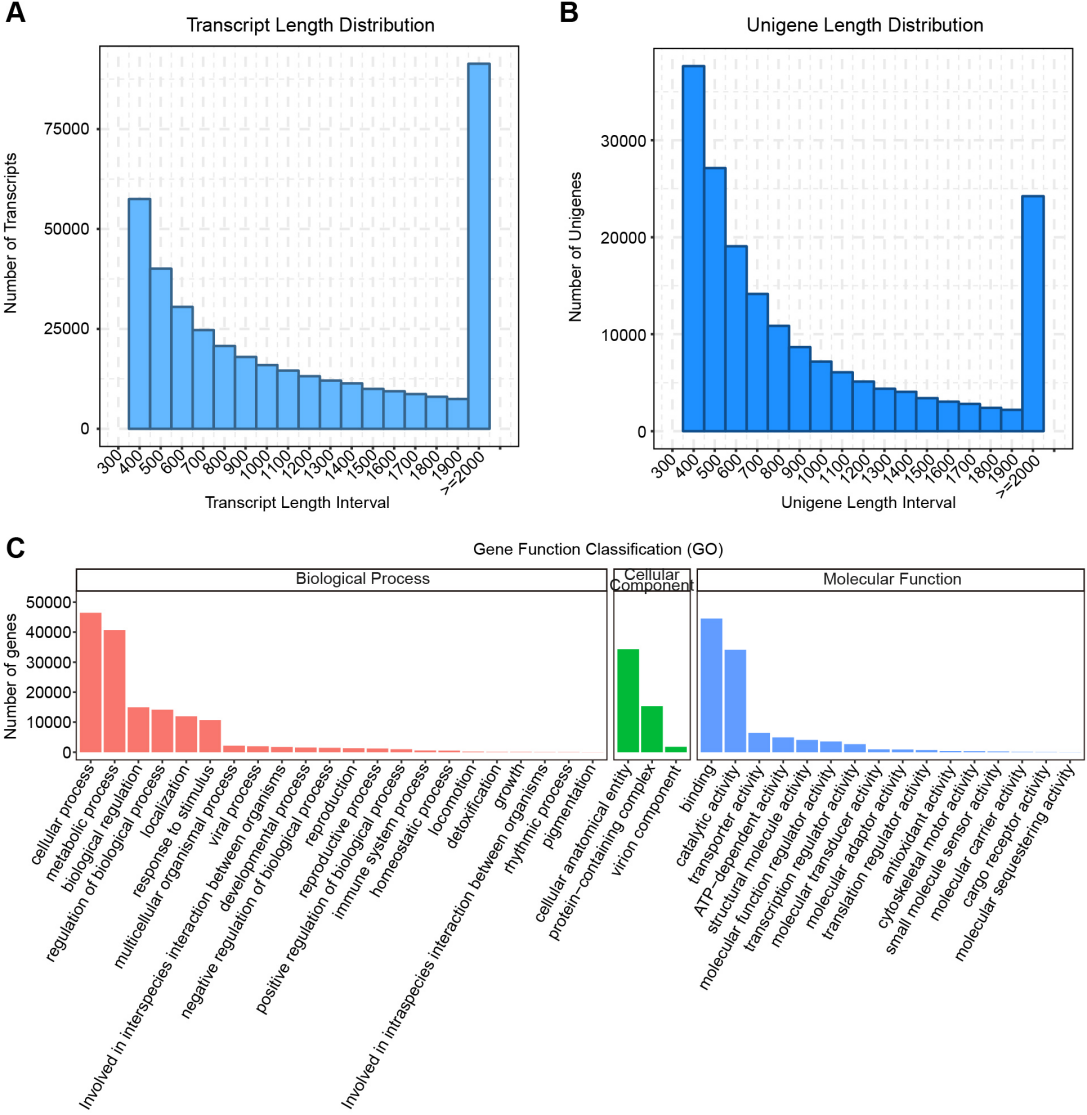
*De novo* assembly results of the amaranth transcriptome. **(A)** Length distribution of assembled transcripts; **(B)** Length distribution of assembled unigenes; **(C)** Gene function classification statistics.

Through Illumina high-throughput sequencing and assembly, we obtained high-quality transcripts and corresponding unigenes for amaranth (**Supplementary Table 2**). The transcript length distribution showed that most transcripts were concentrated in the longer length intervals, indicating good assembly completeness (**Figure 2A**). The unigene length distribution showed a similar trend (**Figure 2B**), further demonstrating the high quality of this transcriptome assembly, capable of covering the complete coding regions of most genes.

To clarify the function of the assembled genes, we compared the unigene sequences of amaranth with seven public databases (**Supplementary Figure 2A**), providing a solid foundation for subsequent differential expression analysis. Specifically, “41.59%”, “44.71%”, and “41.59%” of unigenes were successfully annotated to the GO (Gene Ontology), NR (NCBI non-redundant protein sequences), and Pfam (Protein family) databases, respectively (**Supplementary Figure 2A**). Species distribution analysis of the NR annotation results found that the assembled genes had the highest sequence similarity to the conspecific species “*Amaranthus tricolor*” (37.1%), followed by “*Beta vulgaris subsp. Vulgaris*” (2.3%), etc. (**Supplementary Figure 2B**). This result is consistent with the evolutionary relationships between species, indirectly proving the reliability of the gene annotation.

GO functional classification of the successfully annotated amaranth genes showed that these genes participate widely in various biological processes (**Figure 2C**). In “Biological Process”, highly enriched categories included “cellular process”, “metabolic process”, “response to stimulus”, and “biological regulation”; in “Molecular Function”, genes were mainly concentrated in functions like “binding” and “catalytic activity”; and in “Cellular Component”, they were primarily located in “cell membrane”, “organelle”, and “macromolecular complex”. These classifications indicate that our transcriptome data can comprehensively capture various physiological and molecular processes potentially involved in amaranth’s response to Cd stress.

### Transcriptome data quality assessment and sample relationship analysis

**Figure 3:**
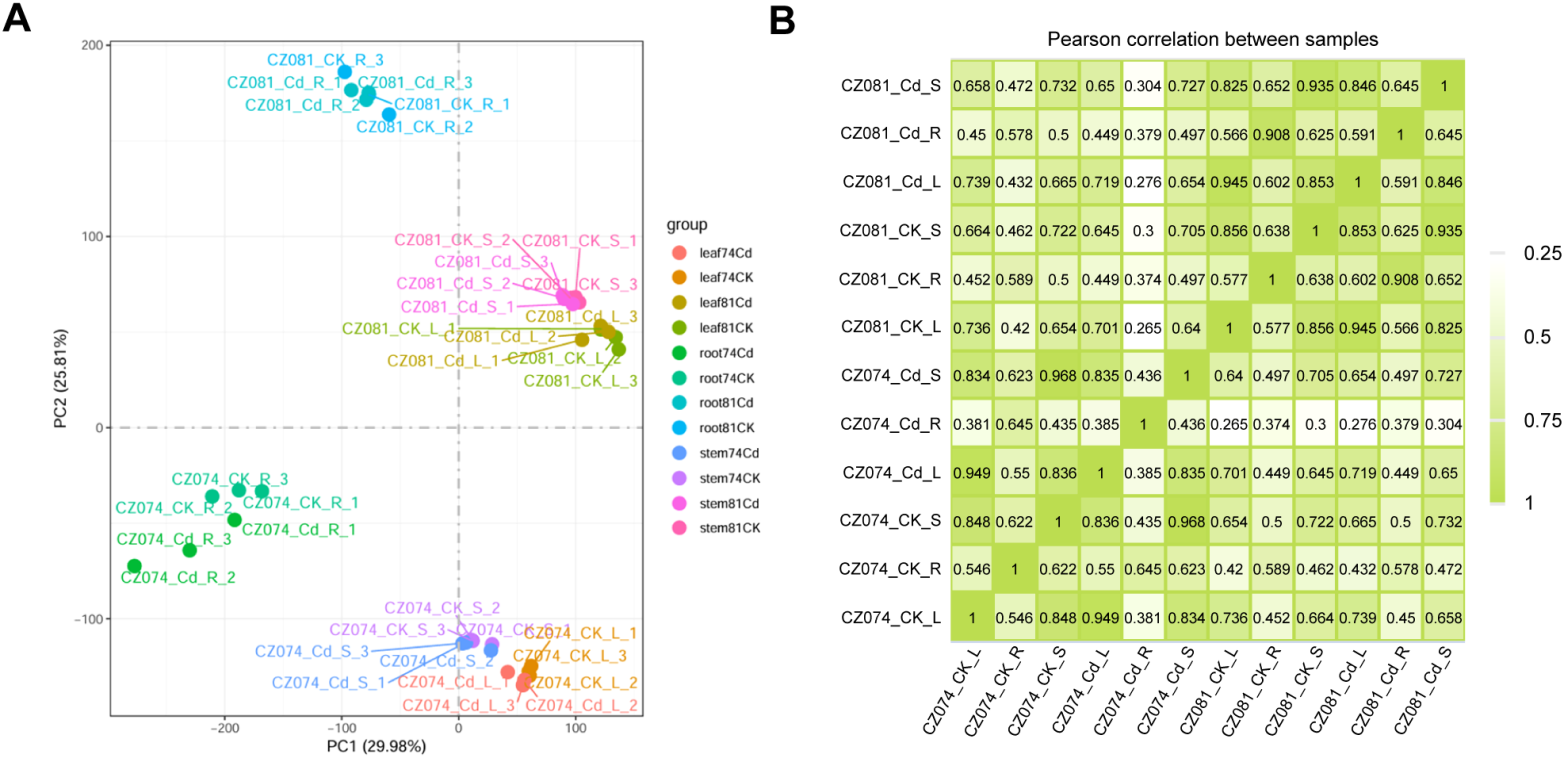
Quality assessment of transcriptome data. **(A)** Principal Component Analysis (PCA) showing the distribution of all samples. PC1 and PC2 explain 29.98% and 25.61% of the total variance, respectively; **(B)** Heatmap of Pearson correlation coefficients between samples. A correlation coefficient closer to 1 indicates higher similarity in expression patterns between samples.

To ensure the reliability of subsequent analyses, we performed strict quality control on the gene expression data of all samples. First, Principal Component Analysis (PCA) visually displayed the overall differences between samples. As shown in **Figure 3A**, principal component 1 (PC1) and principal component 2 (PC2) explained 29.98% and 25.81% of the total variance, respectively. In the two-dimensional space, sample points were clearly separated by “tissue type” (root, stem, leaf), indicating that differences in gene expression patterns between tissues are the largest source of variation. Furthermore, within the same tissue, Cd-treated and control groups showed a certain separation trend, indicating that Cd stress induced significant transcriptome reprogramming in each tissue.

Then, we calculated the Pearson correlation coefficients between all samples (**Figure 3B**). The results showed high positive correlations (correlation coefficients generally > 0.9) between biological replicates under the same treatment conditions, indicating stable experimental operations and good biological reproducibility. Meanwhile, correlation coefficients were significantly lower between different treatment groups and different tissues, suggesting that Cd treatment and organ specificity significantly affected the gene expression profiles.

By plotting the FPKM density distribution for all samples (**Supplementary Figure 3A**), we found that the shapes of all curves highly overlapped and all showed a typical bimodal distribution. This result further demonstrates the consistency of sequencing depth across different samples and the excellent reproducibility of biological replicates, ensuring the accuracy of subsequent differential expression gene screening. Using WGCNA to construct a gene co-expression network, we identified multiple gene modules with distinct expression patterns (**Supplementary Figure 3B**). The module-trait relationship heatmap showed that some modules were highly specifically associated with particular tissues or treatment conditions. For example, some modules (e.g., MEturquoise) might be activated by Cd treatment across all tissues, while others (e.g., MEblue) might respond specifically to Cd stress only in roots. The discovery of these characteristic modules provides important clues for precisely mining the core regulatory pathways of amaranth’s response to Cd stress.

### Analysis of differentially expressed genes in response to Cd stress in amaranth

**Figure 4:**
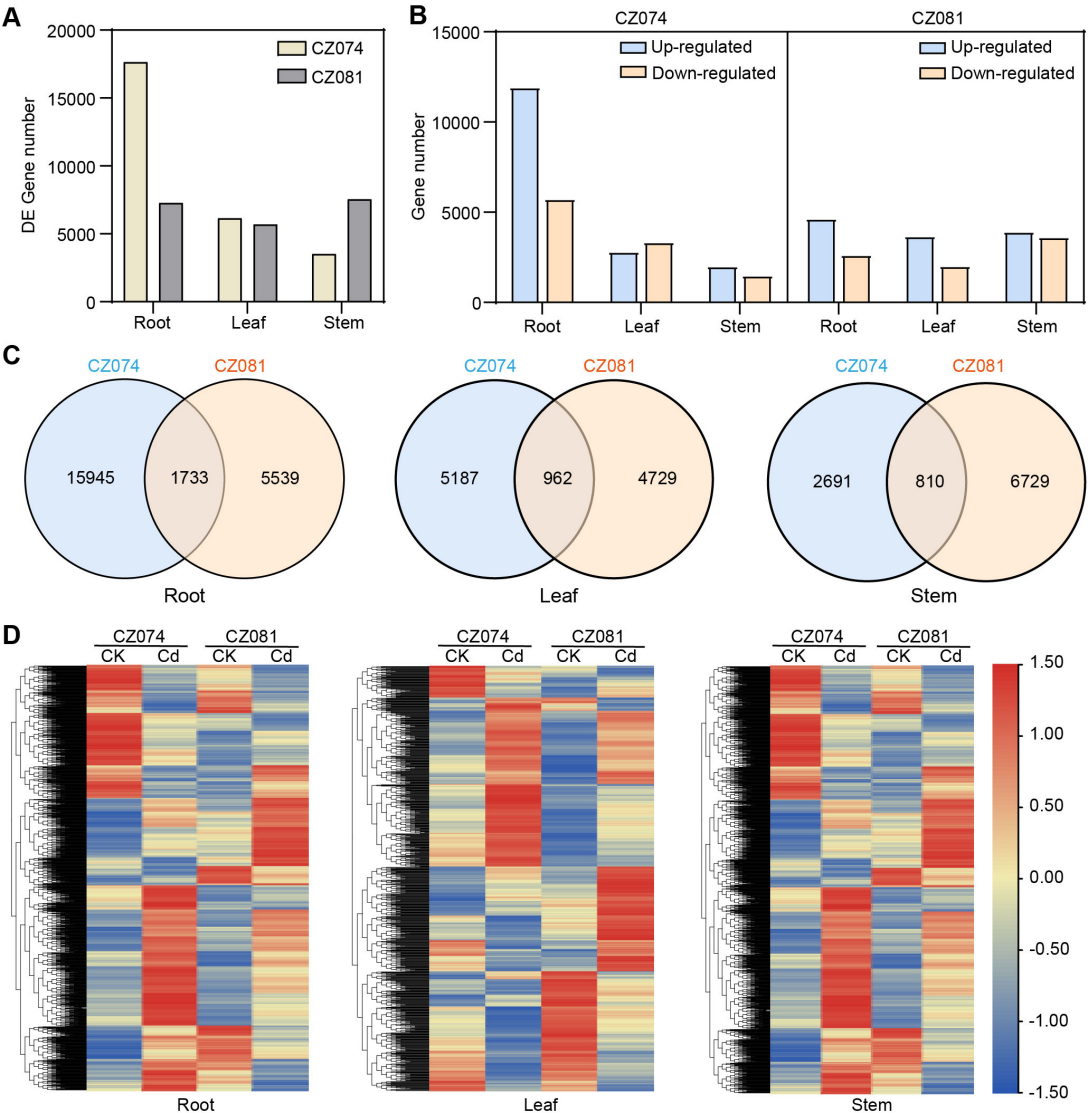
Statistics of differentially expressed genes (DEGs) between two varieties. **(A)** Number of DEGs identified in root, stem, and leaf tissues of CZ074 and CZ081; **(B)** Number of up-regulated and down-regulated DEGs in different tissues of CZ074 and CZ081; **(C)** Venn diagram of common DEGs in root, stem, and leaf tissues between CZ074 and CZ081; **(D)** Heatmap of expression patterns of common DEGs between the two varieties.

To analyze the transcriptional regulatory differences in response to Cd stress between the two amaranth varieties (CZ074 and CZ081), we compared gene expression between Cd-treated and control groups in root, stem, and leaf tissues (**Supplementary Tables 3–8**). First, under 1 mg/L CdCl_2_ stress, a large number of differentially expressed genes (DEGs) were identified in all three tissues of both varieties, but there were significant differences in quantity between varieties (**Figure 4A**). Overall, the total number of DEGs in the tolerant variety CZ074 was much higher than that in the sensitive variety CZ081. This difference was most pronounced in the roots, where the number of DEGs in CZ074 roots (17,641) was 2.4 times that in CZ081 roots (7,272). This indicates that Cd stress induced more extensive transcriptome reprogramming in the tolerant variety.

Further analysis of the up- and down-regulation of DEGs (**Figure 4B**) revealed obvious differences in regulatory patterns between the two varieties. In roots, both cultivars exhibited more up- regulated than down-regulated genes under Cd stress; however, CZ074 showed a relatively higher up-/down-regulated gene ratio than CZ081.This difference suggests that the tolerant variety CZ074 may initiate large-scale gene expression regulation to cope with Cd stress.

Venn diagram analysis of DEGs commonly responsive between the two varieties (**Figure 4C**) identified 1,733, 962, and 810 common DEGs in roots, stems, and leaves, respectively. Statistical analysis of the expression direction of these core gene sets (**Supplementary Figure 4**) found that although they are common response genes in both varieties, their up-/down-regulation patterns still had subtle differences between varieties. For example, in roots, up-regulated genes dominated the common genes in both varieties (CZ074: 1,108 up; CZ081: 1,106 up), but in leaves, the ratio of common up-/down-regulated genes in CZ081 (633 up/329 down) was higher than in CZ074 (541 up/421 down). Analysis of the expression levels of these common DEGs (**Figure 4D**) found that even for the same gene, its expression change-fold (log_2_ FC) in response to Cd stress often differed between the two varieties. Some genes were strongly induced in one variety but only slightly changed in the other. These results indicate that the Cd tolerance of amaranth is not only related to specific gene sets but also depends on the fine-tuning ability of core stress response genes in different tissues. The tolerant variety CZ074 might alleviate Cd toxicity through a more efficient transcriptional regulatory network, thereby maintaining normal growth and development.

### Systemic Cd stress response genes in amaranth

**Figure 5:**
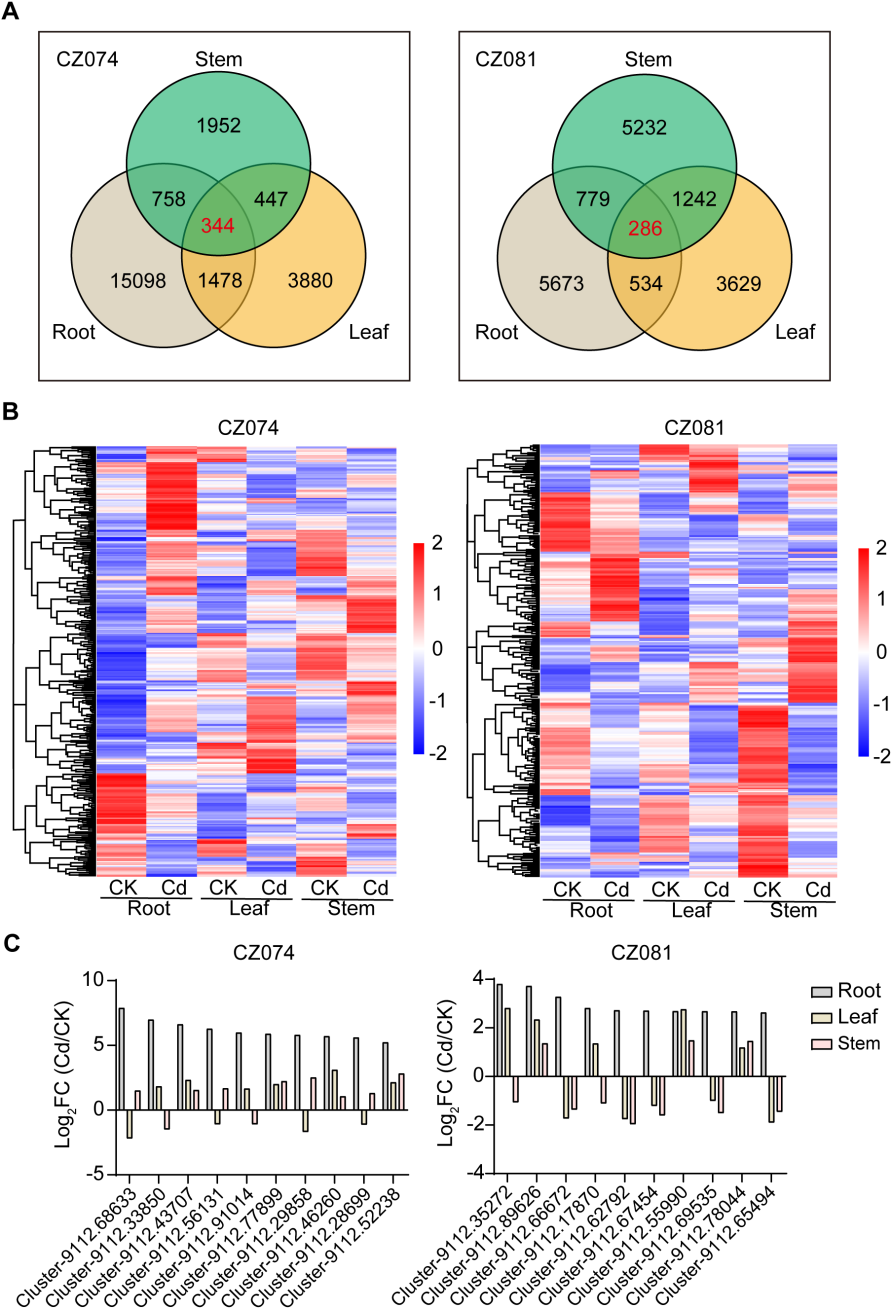
Analysis of cross-tissue differentially expressed genes within each variety. **(A)** Venn diagram analysis of common DEGs in root, stem, and leaf tissues for CZ074 and CZ081; **(B)** Heatmap of expression patterns of common cross-tissue DEGs for CZ074 and CZ081; **(C)** Tissue-specific expression patterns of core cross-tissue responsive genes in tolerant (CZ074, left) and sensitive (CZ081, right) varieties under cadmium stress.

To investigate the synergistic response mechanism of amaranth to Cd stress at the whole plant level, we analyzed genes that were differentially expressed in all three tissues: root, stem, and leaf, i.e., the cross-tissue core response genes. Venn diagram analysis showed (**Figure 5A**) that under Cd stress, the tolerant variety CZ074 had 344 genes commonly differentially expressed in its roots, stems, and leaves; whereas the sensitive variety CZ081 had 286 such genes (**Figure 5A**). This indicates that both varieties possess a core set of genes synergistically regulated throughout the plant body to cope with Cd stress, but the core set of the CZ081 variety is smaller, suggesting its response might be more sluggish.

In-depth analysis of the expression direction of these core genes revealed distinctly different systemic regulatory strategies between the two varieties (**Supplementary Figure 5**). In CZ074, these core genes strongly tended to be up-regulated in the roots (248 up vs 96 down), while they shifted to being predominantly down-regulated in stems and leaves (Stem: 154 up vs 190 down; Leaf: 169 up vs 175 down) (**Supplementary Figure 5**). This pattern depicts an active Cd transport pathway from the “over-reactive” roots to the shoots. In contrast, the expression changes of core genes in CZ081 were more evenly distributed between up- and down-regulation across tissues (root: 134 up vs. 152 down; leaf: 149 up vs. 137 down; stem: 124 up vs. 162 down), without the drastic gene induction seen in CZ074 roots (**Supplementary Figure 5**).

Through further observation of the expression levels of these genes, we found significant differences in the magnitude and patterns of their expression changes between varieties, which may be directly related to their cadmium tolerance. As shown in the **Figure 5B**, the tolerant variety CZ074 exhibited a stronger and more coordinated “root-to-shoot” differential expression pattern in its core cross-tissue genes: most genes showed the most dramatic expression changes in roots (represented by the deepest colors in the heatmap), while changes in stems and leaves were significantly more moderate. In contrast, in the sensitive variety CZ081, the gene set displayed relatively smaller differences in expression changes across tissues, with a more disordered pattern. This difference suggests that CZ074 may achieve more efficient activation of defense mechanisms and reduce aerial damage by concentrating and intensifying core stress responses in roots—the first line of defense. Representative genes selected from these gene sets, along with their specific expression fold changes (log_2_FC) across tissues are listed (**Figure 5C**), providing quantitative evidence for the tissue-specific patterns and varietal differences described above.

### GO functional enrichment analysis of differentially expressed genes in different tissues

**Figure 6:**
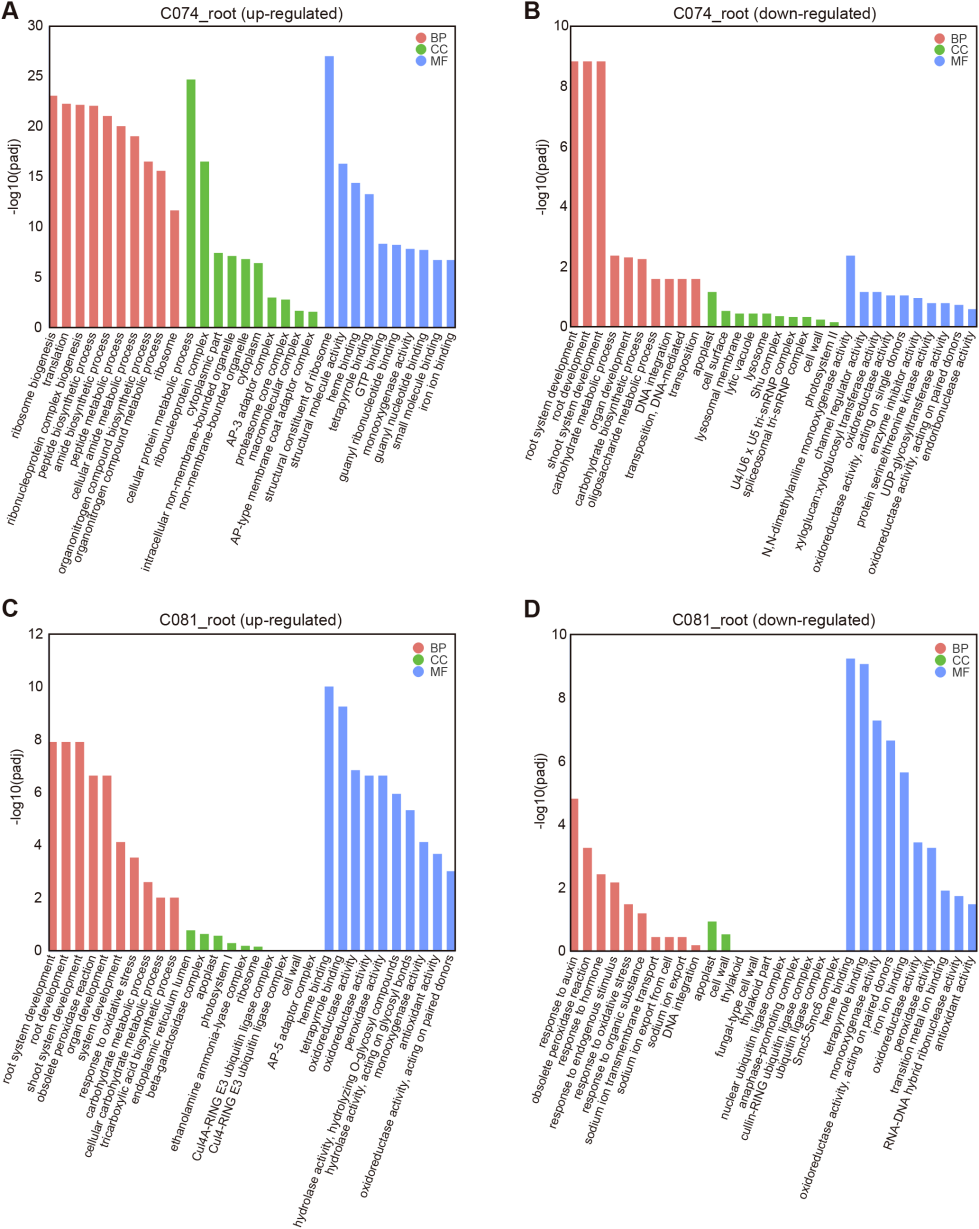
GO functional enrichment analysis of DEGs in roots. **(A, B)** GO Molecular Function enrichment analysis of up-regulated **(A)** and down-regulated **(B)** DEGs in CZ074 roots; **(C, D)** GO Molecular Function enrichment analysis of up-regulated **(C)** and down-regulated **(D)** DEGs in CZ081 roots. The Y-axis represents GO terms, and the X-axis represents the enrichment significance [-log_10_(padj)].

GO enrichment analysis of up- and down-regulated DEGs in the roots of CZ074 and CZ081 revealed significant differences at the molecular function level between the two varieties, closely related to their Cd tolerance phenotypes. For example, among the up-regulated genes in CZ074 roots, the most significantly enriched molecular function terms were ribosome biogenesis, translation, ribonucleoprotein complex biogenesis, and peptide biosynthetic process (**Figure 6A**). This indicates that under Cd stress, CZ074 roots initiated a robust protein synthesis system, enhancing the expression of important proteins to resist Cd stress. This is one of the key molecular mechanisms for its stronger Cd tolerance. In contrast, the enrichment degree of up-regulated genes in CZ081 roots in these biological processes was significantly lower (**Figure 6C**), suggesting its ability to respond to Cd stress might be weaker than CZ074.

Down-regulated genes in CZ081 roots exhibited a very unique enrichment pattern, with the most significant term being “response to auxin” (**Figure 6D**; **Supplementary Tables 9**). The significant down-regulation of this function indicates that Cd stress severely affected the growth and development of CZ081 root cells, potentially involving functional abnormalities in key structures like the cytoskeleton and cell wall, directly leading to disordered root cell function and growth inhibition. In contrast, “response to auxin” was not significantly enriched in down-regulated genes of CZ074 roots (**Figure 6B**), indicating that the tolerant variety successfully maintained cellular structural stability.

To explore the response mechanisms in the shoots under Cd stress, we also performed GO enrichment analysis on DEGs in leaf and stem tissues, finding that their enrichment patterns differed significantly from those in roots, and the two varieties exhibited different adaptation strategies. In CZ074 leaves, up-regulated genes were significantly enriched in functions related to “response to auxin”, while its down-regulated genes were mainly related to processes like “microtubule-based movement”. In CZ081 leaves, neither up- nor down-regulated genes showed significant enrichment in functions related to “response to auxin” or “microtubule-based movement” (**Supplementary Figures 6A–D**).

In the stems of both CZ074 and CZ081, up-regulated genes were significantly enriched in the “response to auxin” function, suggesting smaller functional differences in stems compared to roots and leaves (**Supplementary Figures 6E–H**). In summary, GO enrichment analysis reveals at the molecular function level: the tolerant variety CZ074 can enhance its Cd resistance by regulating the activation and suppression of related pathways in both shoots and roots.

### KEGG pathway enrichment analysis of differentially expressed genes

**Figure 7:**
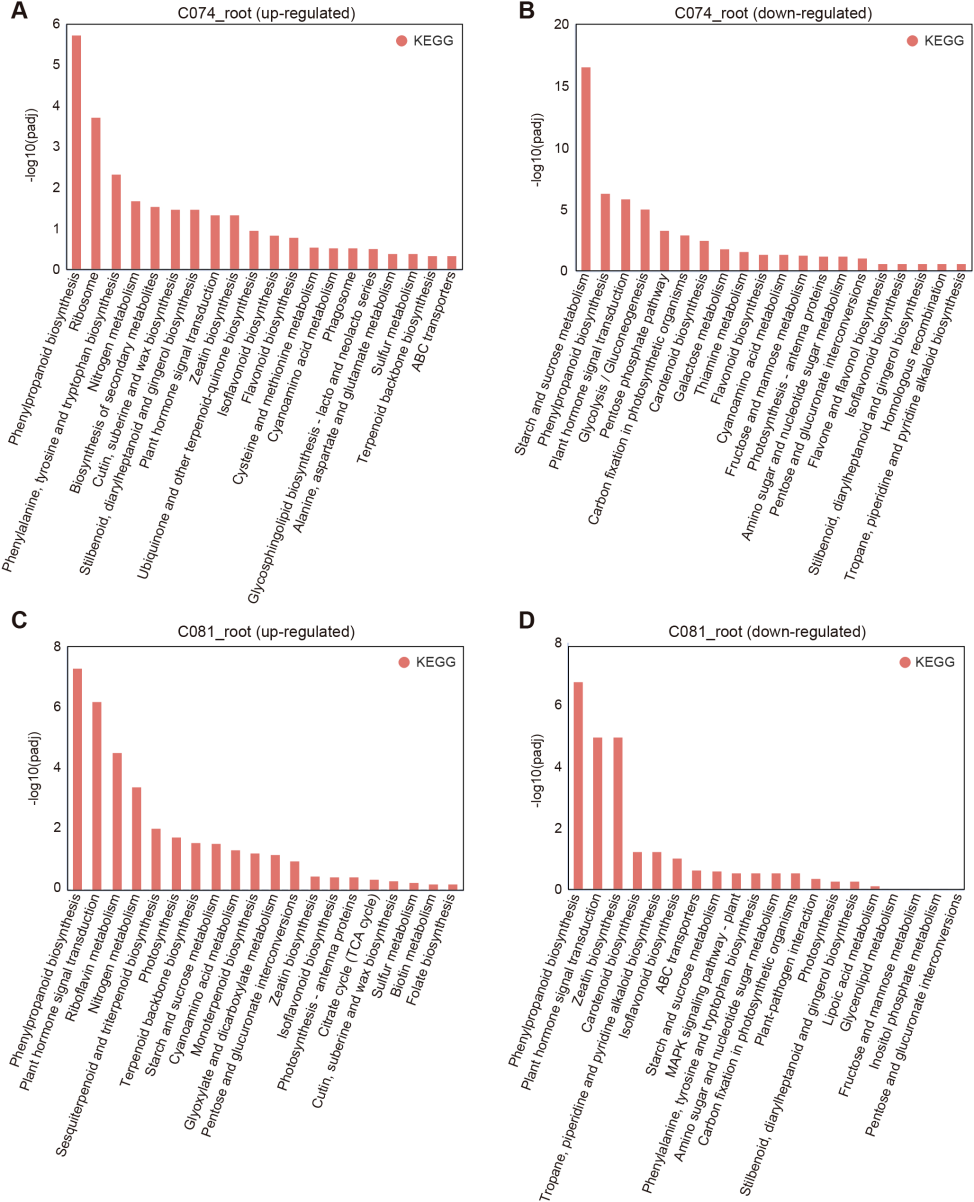
KEGG pathway enrichment analysis of DEGs in roots. **(A, B)** KEGG pathway enrichment analysis of up-regulated **(A)** and down-regulated **(B)** DEGs in CZ074 roots; **(C, D)** KEGG pathway enrichment analysis of up-regulated **(C)** and down-regulated **(D)** DEGs in CZ081 roots. The Y-axis represents KEGG pathways, and the X-axis represents the enrichment significance [-log_10_(padj)].

To analyze the metabolic and signal transduction mechanisms of amaranth’s response to Cd stress at a systems level, we performed KEGG pathway enrichment analysis on DEGs from roots, stems, and leaves of both varieties. The results showed that the responses at the pathway level had both commonalities and distinct characteristics among different varieties and tissues (**Supplementary Table 10**). In the roots, up-regulated genes of both varieties were significantly enriched in the “phenylpropanoid biosynthesis” and “plant hormone signal transduction” pathways (**Figures 7A, C**), indicating that these are core conserved mechanisms for amaranth’s response to Cd stress. Phenylpropanoids are important secondary metabolites in plant stress resistance, involved in cell wall lignification and antioxidation (Ninkuu et al., 2025).

However, there were key differences between varieties: The tolerant variety CZ074 had more abundant and specific uniquely up-regulated pathways, including “cutin, suberine, and wax biosynthesis” closely related to cell wall barrier formation, and “phenylalanine, tyrosine and tryptophan biosynthesis” as well as “biosynthesis of secondary metabolites” related to stress tolerance (**Figure 7A**). Furthermore, the significant up-regulation of the “Ribosome” pathway suggests that CZ074 might support its extensive stress response by enhancing protein synthesis capacity. The sensitive variety CZ081’s up-regulated pathways showed signs of “metabolic disorder”, with “photosynthesis” related pathways abnormally activated in its roots (**Figure 7C**), which might be a manifestation of stress-induced metabolic dysregulation. Simultaneously, its down-regulated genes were significantly enriched in the “MAPK signaling pathway” (**Figure 7D**), which plays a central role in regulating plant stress responses and programmed cell death (Zhang and Zhang, 2022), and its suppression might impair CZ081’s ability to coordinate defense responses.

KEGG analysis of stems and leaves further revealed the response patterns in shoots. In CZ074 leaves, up-regulated genes were enriched in pathways like “betalain biosynthesis” and “zeatin biosynthesis”, related to abiotic stress regulation, to prevent Cd damage (Piotrowska-Niczyporuk et al., 2024; Sabir et al., 2025) (**Supplementary Figure 7**). In CZ074 stems, up-regulated genes might be dedicated to maintaining the stability of “photosynthesis” and related pigment synthesis pathways to ensure energy supply. In contrast, shoots of CZ081 showed down-regulation in pathways more related to “carbohydrate metabolism” (e.g., starch and sucrose metabolism) and “plant hormone signal transduction”, indicating that its energy metabolism and hormone signaling functions were severely disrupted by Cd stress (**Supplementary Figure 7**).

### Cd accumulation distribution characteristics and expression patterns of related regulatory genes

To clarify the physiological and molecular mechanisms underlying the difference in Cd tolerance between the two varieties, we measured Cd accumulation over different time periods and analyzed the expression patterns of key Cd regulatory genes.

**Figure 8:**
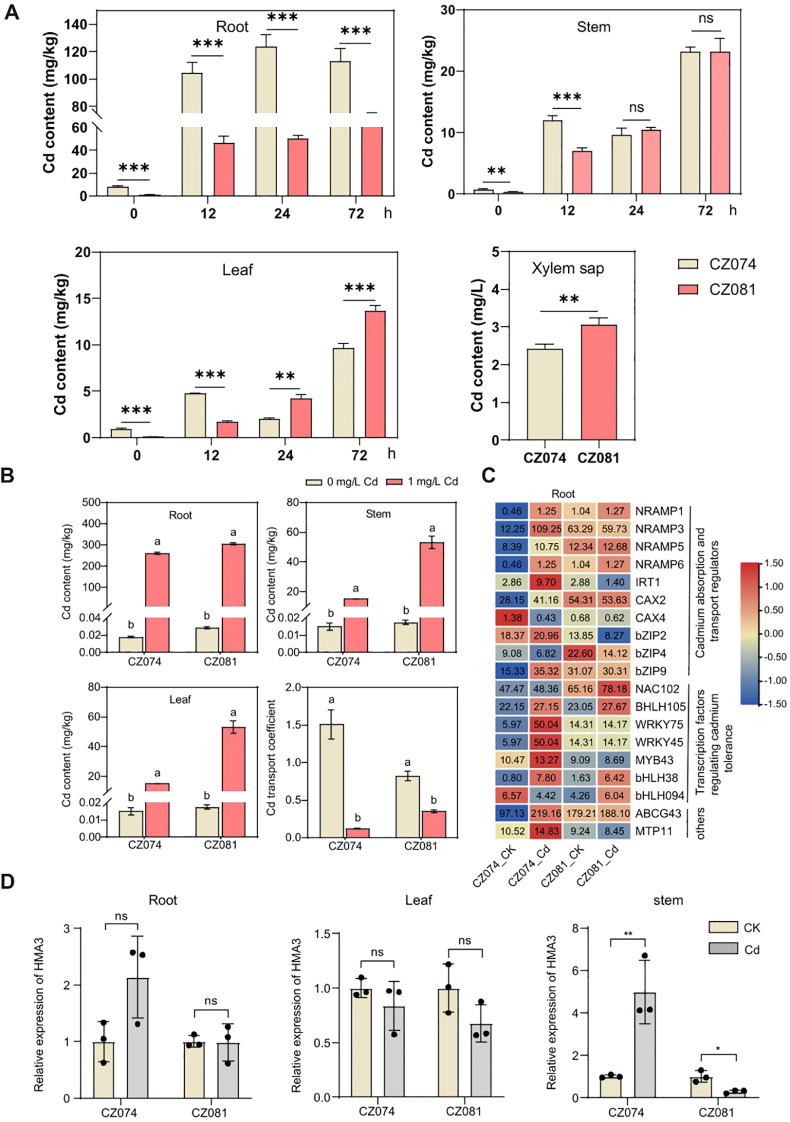
Analysis of cadmium accumulation characteristics and key regulatory gene expression. **(A)** Determination of Cd content in roots, stems, and leaves of the two varieties at 0, 12, 24, and 72 h following Cd treatment (1 mg/L), as well as Cd content in the xylem sap of stems at 72 h post-treatment. Data are presented as mean ± SD. Different lowercase letters above bars indicate significant differences between varieties within the same tissue: *, ** and *** indicate significant differences between treatment and control within the same tissue at *P* < 0.05, *P* < 0.01 and *P* < 0.001 levels, respectively; “ns” indicates not significant (Student’s t-test); **(B)** Determination of Cd content in roots, stems, and leaves of the two varieties on the tenth day after stress and analysis of the Cd transport coefficient.; **(C)** Heatmap of expression of regulators and transcription factors related to Cd absorption and transport in roots; **(D)** Relative expression levels (qRT-PCR validation) of the key Cd transporter gene HMA3 in roots, stems, and leaves.

Time-course analysis showed that Cd accumulation in roots was consistently higher in CZ074 than in CZ081 at all-time points and remained elevated with prolonged treatment. In contrast, in stems and leaves, CZ074 exhibited higher Cd levels than CZ081 at early stages (12–24 h), whereas at 72 h, Cd concentrations in CZ081 gradually increased and exceeded those in CZ074 (**Figure 8A**). These results indicate distinct temporal patterns of Cd distribution between the two cultivars. Consistent with this observation, Cd concentration in xylem sap was significantly lower in CZ074 than in CZ081 (**Figure 8A**), suggesting reduced root-to-shoot Cd transport in the tolerant cultivar. In contrast, the higher Cd levels in xylem sap of CZ081 are associated with continued Cd accumulation in aboveground tissues at later stages.

Determination of Cd content in various tissues under 1 mg/L CdCl_2_ treatment showed that the roots were the main Cd accumulation organ for both varieties (**Figure 8B**). However, the Cd transport coefficient from roots to shoots was significantly higher in the tolerant variety CZ074 than in the sensitive variety CZ081 (**Figure 8B**). This suggests that CZ074 might alleviate the direct toxicity of Cd to the root system by translocating more Cd from the stress-sensitive underground roots to the shoots, representing an important tolerance strategy.

We analyzed the expression of a series of key genes involved in Cd absorption, transport, and detoxification in the roots (**Supplementary Table 11**). The heatmap shows that under Cd stress, the expression up-regulation amplitude of most key regulatory genes in the roots of the tolerant variety CZ074 was significantly greater than in CZ081 (**Figure 8C**). Cd absorption and transport regulators such as the transporter genes *IRT1* and *CAX2*, and *NRAMP3* and *NRAMP6*, which may play a role in long-distance Cd transport (He et al., 2017; Wang et al., 2019; Jia et al., 2022; Liu et al., 2025), were more strongly induced in CZ074 roots (**Figure 8C**).

Cd tolerance transcription factors including multiple members of the *bZIP*, *NAC102*, *BHLH105*, and *WRKY* families (Han et al., 2023; Li et al., 2023a; Lu et al., 2024; Meng et al., 2024), also showed more significant up-regulation in CZ074 roots (**Figure 8C**). These transcription factors are core hubs regulating plant heavy metal stress responses, and their strong activation indicated that CZ074 initiated a broader and more efficient defense gene network.

We further analyzed the expression of the key vacuolar sequestration transporter gene *HMA3* (Liu et al., 2017; Park and Ahn, 2017; Liao et al., 2023). The results showed that in root and stem tissue, the Cd-induced expression level of *HMA3* was significantly higher in CZ074 than in CZ081 (**Figure 8D**). This result is consistent with the overall expression pattern of the aforementioned transporter genes, further supporting that CZ074 may possess a stronger Cd compartmentalization capacity, thereby fixing it in the vacuoles of tissues like stems, preventing its transfer to active physiological sites (e.g., leaves), and ultimately achieving higher overall tolerance.

We also analyzed the expression levels of key Cd regulatory genes in leaves and stems (**Supplementary Figure 8A**). In leaves, the expression of multiple genes in both varieties changed after Cd treatment, but the regulatory patterns differed between varieties. CZ081 showed higher induced expression levels for several genes. For example, the transporter genes *NRAMP1*, *NRAMP3*, *NRAMP5* and *NRAMP6*, responsible for Cd efflux or sequestration (Kanwal et al., 2024), maintained relatively high expression levels in CZ081. Conversely, the expression of these genes decreased after Cd treatment in CZ074.

The gene expression pattern in stem tissue was more complex (**Supplementary Figure 8B**). Notably, the vacuolar Cd transporter genes *CAX2* and *CAX4* were more strongly induced by Cd in CZ081 stems. However, the expression levels of *NRAMP1* and *NRAMP6* in CZ081 stems were significantly lower compared to CZ074. Transcription factors NAC102, bHLH35, bHLH094, etc., also showed varietal differences in expression in stems, suggesting their potential involvement in the specific regulation of Cd response in stem tissues.

Together, our experimental and transcriptomic data converge on a putative model in which Cd tolerance in CZ074 is driven by preferential sequestration in roots and strictly limited translocation to the shoot.

## Discussion

This study systematically compared the response mechanisms to Cd stress in two amaranth varieties (tolerant CZ074 and sensitive CZ081) by integrating physiological phenotypes, Cd accumulation characteristics, and multi-tissue transcriptome data. Our research not only confirmed significant differences in Cd tolerance between the varieties but, more importantly, revealed the intrinsic reasons for these differences at the transcriptional regulatory level, depicting a more refined Cd tolerance regulatory network from perception and signal transduction to physiological execution.

### Active global transcriptional reprogramming as the foundation of Cd tolerance

The observed phenomenon of the tolerant variety having more differentially expressed genes provides an interesting perspective for understanding plant stress response strategies. The traditional “energy-saving” model suggests that precise regulation of a small number of key genes is a hallmark of efficient tolerance (Raza et al., 2025). However, our results support an alternative “comprehensive mobilization” adaptive strategy. Under severe stress, activating broader transcriptome reprogramming may imply stronger stress perception and signal transduction capabilities, thereby mobilizing more diverse physiological and biochemical pathways for coordinated defense (Himanen and Sistonen, 2019; Han et al., 2020). Although this large-scale investment consumes energy, it may build a more robust defense system. Conversely, the limited number of DEGs in CZ081 might indicate obstructed stress signal pathways or insufficient function of key transcriptional regulatory elements, preventing it from effectively coordinating a defense response of sufficient scale, thus manifesting sensitivity at the physiological level (Yin et al., 2025). Therefore, the “quantity” of DEGs itself may be as important as their “quality” (i.e., functional coordination and specificity), serving as a key indicator for measuring plant stress response capacity (Zhao et al., 2018).

### Roots: trade-off between defense and growth, and maintenance of metabolic homeostasis

Roots are the primary site for perceiving and responding to Cd stress (Lux et al., 2011). Our results indicate that CZ074 adopts an active physiological trade-off strategy to cope with stress. The strong up-regulation of genes related to functions like oxidoreductase activity in its roots coexists with the down-regulation of genes related to cell division and growth, consistent with the classic stress adaptation theory where plants reallocate resources from growth to defense (Wang et al., 2020; Li et al., 2023c). Meanwhile, the specific induction of the cutin, suberine, and wax biosynthesis pathway suggests that CZ074 might enhance the lignification and suberization of the cell wall to construct a physical barrier, limiting Cd ion entry into the symplast (Philippe et al., 2020).

In contrast, CZ081’s response pointed to the disruption of metabolic homeostasis. The abnormal activation of photosynthesis-related genes in its roots is a typical sign of metabolic disorder (Zhang et al., 2020; Chauhan et al., 2023). More critically, the significant down-regulation of the MAPK signaling pathway – a core pathway regulating stress responses and programmed cell death – might severely impair CZ081’s ability to coordinate downstream defense gene expression, leading to the failure of its coping strategy (Zhang and Zhang, 2022; Manna et al., 2023).

### The decisive role of the core transcription factor network

The gene expression heatmap clearly showed that a whole set of transcription factors (such as *bZIP*, bHLH, WRKY family members) involved in Cd tolerance regulation were more strongly activated in CZ074 roots. These transcription factors are core hubs regulating plant heavy metal stress responses (Liu et al., 2024; Zhang and Lu, 2024). Their coordinated up-regulation likely drives the expression of a series of downstream defense genes, including those for metal chelation, antioxidant defense, and cell wall modification, thus constituting the transcriptional basis for CZ074’s strong Cd tolerance (Li et al., 2023b). NRAMP family genes, as core Cd uptake transporters, were also highly induced in CZ074 roots, which might be a main reason for CZ074’s higher Cd transport coefficient (Kanwal et al., 2024; Arif et al., 2025).

### Temporal uncoupling of cadmium uptake and translocation defines varietal tolerance strategies

Our time-resolved analysis of Cd distribution unveils a critical, stage-dependent divergence in how the two varieties manage the toxic metal. Both varieties initially absorb Cd into their roots, but their subsequent fates diverge dramatically. The tolerant CZ074 demonstrates a strategy of progressive root sequestration: after an initial rise, shoot Cd levels plateau or even decline by 72 hours, concomitant with significantly lower Cd concentration in the xylem sap. This indicates the establishment of an effective root-based barrier that limits further loading into the vascular system. In contrast, the sensitive CZ081 fails to exert comparable control over root-to-shoot Cd transport. Cd concentrations in stems and leaves of CZ081 continue to increase throughout the treatment period, accompanied by consistently higher Cd levels in the xylem sap. Together, these observations indicate insufficient regulation of xylem loading and long-distance transport, resulting in progressive Cd movement from roots to aboveground tissues over time (Baruah et al., 2023; Zhang et al., 2024a). The transcriptomic data further support this model. In CZ074, the early and strong induction of root vacuolar sequestration genes, such as *HMA3*, provides a mechanistic basis for its enhanced capacity to sequester Cd within root cells. In contrast, the weaker induction of these transporters in CZ081 may underlie its reduced ability to retain Cd in root vacuoles, thereby facilitating continued Cd translocation to aboveground tissues, leading to the observed sustained translocation (Liu et al., 2017; Park and Ahn, 2017). This temporal perspective highlights tolerance as a dynamic process that involves effective confinement of the toxin to roots over time, rather than a static trait.

### Integration of transport physiology and transcriptional control: toward a predictive model of Cd partitioning

The combined physiological and molecular data allow us to propose an integrated model for Cd partitioning governed by orchestrated transporter expression. In CZ074, a coordinated transcriptional program is activated: high-affinity root uptake systems (e.g., *IRT1*, *NRAMPs*) are induced to potentially sequester Cd in root apoplast or symplast, while simultaneously, genes mediating xylem loading or root-to-shoot translocation are more tightly regulated or counterbalanced by potent sequestration mechanisms (e.g., *HMA3*) (Liu et al., 2024; Zhang and Lu, 2024). This creates a scenario where root absorption capacity is high, but the transport to the shoot is restricted. Conversely, in CZ081, the transcriptional response appears less coordinated. While some uptake genes are induced, the key sequestration machinery is under-activated, and the regulatory check on translocation may be absent or inefficient. This imbalance results in Cd being absorbed but not adequately contained, leading to its continuous flow into the shoot. The strong, yet seemingly detrimental, up-regulation of several *NRAMP* and *CAX* genes in CZ081 shoots might be a futile compensatory response to this influx, further disrupting leaf ion homeostasis (Tao and Lu, 2022; Zheng et al., 2023). This integrated view suggests that future efforts to manipulate Cd distribution in plants should focus not on single transporters, but on the regulatory nodes that synchronize the entire uptake-sequestration-translocation network. Transcription factors like the strongly CZ074-upregulated *NAC102* and *bZIPs* are prime candidates for being such master regulators.

Cd tolerance in CZ074 is achieved through a coordinated dual mechanism: an early activation of root-based absorption and detoxification gene networks, followed by the establishment of effective root-to-shoot transport barriers and vacuolar sequestration. This strategy restricts Cd translocation to physiologically active aerial tissues, thereby mitigating overall phytotoxicity. By contrast, the sensitive cultivar CZ081 lacks this coordinated regulation, resulting in less root retention, greater Cd accumulation in stems, and more severe growth inhibition. These findings highlight the importance of temporal and tissue-specific regulation in determining varietal differences in Cd tolerance.

## Conclusion

In summary, we propose a working model for Cd tolerance in amaranth: the tolerant variety CZ074 activates a precise transcriptional regulatory network led by key transcription factors, strengthens antioxidant and physical barrier defenses in the roots, for safe translocation and fixation, ultimately achieving an optimal balance between growth and defense at the organ and whole-plant levels. In contrast, the sensitive variety CZ081, due to suppressed core signaling pathways and metabolic disorder, cannot establish this coordinated network, ultimately leading to severely impaired growth. The key transporter proteins (e.g., HMA3, CAXs, ABCG43) and transcription factors (e.g., NAC102, bZIPs) identified in this study provide valuable candidate gene resources for future genetic engineering to improve crop Cd tolerance or for screening plant materials for phytoremediation of Cd-contaminated soils. Subsequent work could involve functional validation of these core genes to further reveal their specific roles in Cd tolerance in amaranth. 

## Data Availability

The datasets presented in this study can be found in online repositories. The names of the repository/repositories and accession number(s) can be found in the article/**Supplementary Material**.

## References

[B1] Arif M. Abbas H. Mahmood N. Uzair M. Manzoor M. A. Tung S. A. . (2025). Genome-Wide analysis of the NRAMP gene family in Arabidopsis thaliana: identification, expression and response to multiple heavy metal stresses and phytohormones. BMC Plant Biol. 25, 1305. doi: 10.1186/s12870-025-07396-8, PMID: 41044507 PMC12495693

[B2] Aslam M. M. Okal E. J. Waseem M. (2023). Cadmium toxicity impacts plant growth and plant remediation strategies. Plant Growth Regul. 99, 397–412. doi: 10.1007/s10725-022-00917-7

[B3] Baruah N. Gogoi N. Roy S. Bora P. Chetia J. Zahra N. . (2023). Phytotoxic responses and plant tolerance mechanisms to cadmium toxicity. J. Soil Sci. Plant Nutr. 23, 4805–4826. doi: 10.1007/s42729-023-01525-8

[B4] Chauhan J. Prathibha M. D. Singh P. Choyal P. Mishra U. N. Saha D. . (2023). Plant photosynthesis under abiotic stresses: Damages, adaptive, and signaling mechanisms. Plant Stress 10, 100296. doi: 10.1016/j.stress.2023.100296

[B5] García De La Torre V. S. Coba De La Peña T. Pueyo J. J. Lucas M. M. (2021). Cadmium-tolerant and -sensitive cultivars identified by screening of medicago truncatula germplasm display contrasting responses to cadmium stress. Front. Plant Sci. 12. doi: 10.3389/fpls.2021.595001, PMID: 33777061 PMC7991585

[B6] Genchi G. Sinicropi M. S. Lauria G. Carocci A. Catalano A. (2020). The effects of cadmium toxicity. Int. J. Environ. Res. Public Health 17. doi: 10.3390/ijerph17113782, PMID: 32466586 PMC7312803

[B7] Guo J. Qin S. Rengel Z. Gao W. Nie Z. Liu H. . (2019). Cadmium stress increases antioxidant enzyme activities and decreases endogenous hormone concentrations more in Cd-tolerant than Cd-sensitive wheat varieties. Ecotoxicol. Environ. Saf. 172, 380–387. doi: 10.1016/j.ecoenv.2019.01.069, PMID: 30731269

[B8] Haider F. U. Liqun C. Coulter J. A. Cheema S. A. Wu J. Zhang R. . (2021). Cadmium toxicity in plants: Impacts and remediation strategies. Ecotoxicol. Environ. Saf. 211, 111887. doi: 10.1016/j.ecoenv.2020.111887, PMID: 33450535

[B9] Han G. H. Huang R. N. Hong L. H. Xu J. X. Hong Y. G. Wu Y. H. . (2023). The transcription factor NAC102 confers cadmium tolerance by regulating WAKL11 expression and cell wall pectin metabolism in Arabidopsis. J. Integr. Plant Biol. 65, 2262–2278. doi: 10.1111/jipb.13557, PMID: 37565550

[B10] Han N. C. Kelly P. Ibba M. (2020). Translational quality control and reprogramming during stress adaptation. Exp. Cell Res. 394, 112161. doi: 10.1016/j.yexcr.2020.112161, PMID: 32619498

[B11] He F. Liu Q. Zheng L. Cui Y. Shen Z. Zheng L. (2015). RNA-seq analysis of rice roots reveals the involvement of post-transcriptional regulation in response to cadmium stress. Front. Plant Sci. 6, 2015. doi: 10.3389/fpls.2015.01136, PMID: 26734039 PMC4685130

[B12] He X. L. Fan S. K. Zhu J. Guan M. Y. Liu X. X. Zhang Y. S. . (2017). Iron supply prevents Cd uptake in Arabidopsis by inhibiting IRT1 expression and favoring competition between Fe and Cd uptake. Plant Soil 416, 453–462. doi: 10.1007/s11104-017-3232-y

[B13] Himanen S. V. Sistonen L. (2019). New insights into transcriptional reprogramming during cellular stress. J. Cell Sci. 132. doi: 10.1242/jcs.238402, PMID: 31676663

[B14] Hu Y. He R. Mu X. Zhou Y. Li X. Wang H. . (2025). Cadmium toxicity in plants: from transport to tolerance mechanisms. Plant Signaling Behav. 20, 2544316. doi: 10.1080/15592324.2025.2544316, PMID: 40843968 PMC12377090

[B15] Hunková J. Lisinovičová M. Lancíková V. Szabóová M. Kačírová J. Mistríková V. . (2024). A comparative analysis of heavy metal stress responses in different grain amaranth cultivars. Plant Stress 14, 100619. doi: 10.1016/j.stress.2024.100619

[B16] Jia H. Yin Z. Xuan D. Lian W. Han D. Zhu Z. . (2022). Mutation of NtNRAMP3 improves cadmium tolerance and its accumulation in tobacco leaves by regulating the subcellular distribution of cadmium. J. Hazard. Mater. 432, 128701. doi: 10.1016/j.jhazmat.2022.128701, PMID: 35313160

[B17] Kanwal F. Riaz A. Ali S. Zhang G. (2024). NRAMPs and manganese: Magic keys to reduce cadmium toxicity and accumulation in plants. Sci. Tot. Environ. 921, 171005. doi: 10.1016/j.scitotenv.2024.171005, PMID: 38378068

[B18] Lancíková V. Tomka M. Žiarovská J. Gažo J. Hricová A. (2020). Morphological Responses and Gene Expression of Grain Amaranth (Amaranthus spp.) Growing under Cd. Plants (Basel). 9, 572. doi: 10.3390/plants9050572, PMID: 32365842 PMC7285102

[B19] Li F. Deng Y. Liu Y. Mai C. Xu Y. Wu J. . (2023a). Arabidopsis transcription factor WRKY45 confers cadmium tolerance via activating PCS1 and PCS2 expression. J. Hazard. Mater. 460, 132496. doi: 10.1016/j.jhazmat.2023.132496, PMID: 37703737

[B20] Li Y. Ding L. Zhou M. Chen Z. Ding Y. Zhu C. (2023). Transcriptional regulatory network of plant cadmium stress response. Int. J. Mol. Sci. 24, 4378. doi: 10.3390/ijms24054378, PMID: 36901809 PMC10001906

[B21] Liao C. Li Y. Wu X. Wu W. Zhang Y. Zhan P. . (2023). ZmHMA3, a member of the heavy-metal-transporting ATPase family, regulates cd and zn tolerance in maize. Int. J. Mol. Sci. 24. doi: 10.3390/ijms241713496, PMID: 37686302 PMC10487686

[B22] Liu X.-Q. Liu H. Fu M.-J. Zhang L.-W. Yin S.-F. Tang Z. . (2025). The cation/H+ exchanger OsCAX2 is involved in cadmium uptake and contributes to differential grain cadmium accumulation between Indica and Japonica rice. J. Hazard. Mater. 487, 137252. doi: 10.1016/j.jhazmat.2025.137252, PMID: 39842113

[B23] Liu J. Ma J. He C. Li X. Zhang W. Xu F. . (2013). Inhibition of cadmium ion uptake in rice (Oryza sativa) cells by a wall-bound form of silicon. New Phytol. 200, 691–699. doi: 10.1111/nph.12494, PMID: 24102436

[B24] Liu C. Wen L. Cui Y. Ahammed G. J. Cheng Y. (2024). Metal transport proteins and transcription factor networks in plant responses to cadmium stress. Plant Cell Rep. 43, 218. doi: 10.1007/s00299-024-03303-x, PMID: 39153039

[B25] Liu H. Zhao H. Wu L. Liu A. Zhao F.-J. Xu W. (2017). Heavy metal ATPase 3 (HMA3) confers cadmium hypertolerance on the cadmium/zinc hyperaccumulator Sedum plumbizincicola. New Phytol. 215, 687–698. doi: 10.1111/nph.14622, PMID: 28574163

[B26] Lu Z. Yu M. Han X. Qiao G. Xu J. Wu L. . (2024). SpbZIP60 confers cadmium tolerance by strengthening the root cell wall compartmentalization in Sedum plumbizincicola. J. Hazard. Mater. 480, 135936. doi: 10.1016/j.jhazmat.2024.135936, PMID: 39321478

[B27] Lux A. Martinka M. Vaculík M. White P. J. (2011). Root responses to cadmium in the rhizosphere: a review. J. Exp. Bot. 62, 21–37. doi: 10.1093/jxb/erq281, PMID: 20855455

[B28] Manna M. Rengasamy B. Sinha A. K. (2023). Revisiting the role of MAPK signalling pathway in plants and its manipulation for crop improvement. Plant Cell Environ. 46, 2277–2295. doi: 10.1111/pce.14606, PMID: 37157977

[B29] Meng Y. Li M. Guo Z. Chen J. Wu J. Xia Z. (2024). The transcription factor ZmbHLH105 confers cadmium tolerance by promoting abscisic acid biosynthesis in maize. J. Hazard. Mater. 480, 135826. doi: 10.1016/j.jhazmat.2024.135826, PMID: 39270588

[B30] Mukuwapasi B. Mavengahama S. Gerrano A. S. (2024). Grain amaranth: A versatile untapped climate-smart crop for enhancing food and nutritional security. Discov. Agric. 2, 44. doi: 10.1007/s44279-024-00057-8

[B31] Mushtaq G. Agrawal S. Kushwah A. Kumar A. Lone R. (2025). Cadmium toxicity in plants and its remediation management: A review. Plant Stress 16, 100894. doi: 10.1016/j.stress.2025.100894

[B32] Ninkuu V. Aluko O. O. Yan J. Zeng H. Liu G. Zhao J. . (2025). Phenylpropanoids metabolism: recent insight into stress tolerance and plant development cues. Front. Plant Sci. 16, 2025. doi: 10.3389/fpls.2025.1571825, PMID: 40641862 PMC12242330

[B33] Park W. Ahn S.-J. (2017). HMA3 is a key factor for differences in Cd- and Zn-related phenotype between Arabidopsis Ws and Col-0 ecotypes. Plant Biotechnol. Rep. 11, 209–218. doi: 10.1007/s11816-017-0447-6

[B34] Philippe G. Sørensen I. Jiao C. Sun X. Fei Z. Domozych D. S. . (2020). Cutin and suberin: assembly and origins of specialized lipidic cell wall scaffolds. Curr. Opin. Plant Biol. 55, 11–20. doi: 10.1016/j.pbi.2020.01.008, PMID: 32203682

[B35] Piotrowska-Niczyporuk A. Bonda-Ostaszewska E. Bajguz A. (2024). Mitigating effect of trans-zeatin on cadmium toxicity in desmodesmus armatus. Cells 13, 686. doi: 10.3390/cells13080686, PMID: 38667301 PMC11049045

[B36] Rani A. Kumar A. Lal A. Pant M. (2014). Cellular mechanisms of cadmium-induced toxicity: a review. Int. J. Environ. Health Res. 24, 378–399. doi: 10.1080/09603123.2013.835032, PMID: 24117228

[B37] Raza A. Li Y. Prakash C. S. Hu Z. (2025). Panomics to manage combined abiotic stresses in plants. Trends Plant Sci. 30, 1079–1084. doi: 10.1016/j.tplants.2025.03.001, PMID: 40148151

[B38] Sabir I. A. Manzoor M. A. Khan I. Hu X. Chen J. Qin Y. (2025). Emerging trends in secondary metabolite research in caryophyllales: betalains and their roles in plant adaptation and defense mechanisms. J. Agric. Food Chem. 73, 2249–2265. doi: 10.1021/acs.jafc.4c10283, PMID: 39818758

[B39] Tao J. Lu L. (2022). Advances in genes-encoding transporters for cadmium uptake, translocation, and accumulation in plants. Toxics 10, 411. doi: 10.3390/toxics10080411, PMID: 35893843 PMC9332107

[B40] Vitelli V. Giamborino A. Bertolini A. Saba A. Andreucci A. (2024). Cadmium stress signaling pathways in plants: molecular responses and mechanisms. Curr. Issues Mol. Biol. 46, 6052–6068. doi: 10.3390/cimb46060361, PMID: 38921032 PMC11202648

[B41] Wang C. Chen X. Yao Q. Long D. Fan X. Kang H. . (2019). Overexpression of TtNRAMP6 enhances the accumulation of Cd in Arabidopsis. Gene 696, 225–232. doi: 10.1016/j.gene.2019.02.008, PMID: 30769144

[B42] Wang H. Xu Y. Zhang Z. Zhang G. Tan C. Ye L. (2024). Development and application of transcriptomics technologies in plant science. Crop Des. 3, 100057. doi: 10.1016/j.cropd.2024.100057

[B43] Wang Q. Zeng X. Song Q. Sun Y. Feng Y. Lai Y. (2020). Identification of key genes and modules in response to Cadmium stress in different rice varieties and stem nodes by weighted gene co-expression network analysis. Sci. Rep. 10, 9525. doi: 10.1038/s41598-020-66132-4, PMID: 32533096 PMC7293223

[B44] Yang Q. Li Z. Lu X. Duan Q. Huang L. Bi J. (2018). A review of soil heavy metal pollution from industrial and agricultural regions in China: Pollution and risk assessment. Sci. Tot. Environ. 642, 690–700. doi: 10.1016/j.scitotenv.2018.06.068, PMID: 29909337

[B45] Yang X. Wang X. Zhang X. Wu D. Cheng Y. Wang Y. . (2025). Full-length transcriptome assembly and RNA-Seq integration of diploid and tetraploid ryegrass to investigate differences in cd uptake and accumulation among ryegrass with different ploidy levels. BMC Genomics 26, 128. doi: 10.1186/s12864-025-11325-2, PMID: 39930350 PMC11812225

[B46] Yildirim E. Agar G. Ors S. Yuksel E. Aydin M. Ekinci M. (2023). Growth, physiological, biochemical and DNA methylation responses to cadmium stress of bean (phaseolus vulgaris L) grown under different irrigation levels. Plant Growth Regul. 101, 537–556. doi: 10.1007/s10725-023-01039-4

[B47] Yin H. Duo H. Li S. Qin D. Xie L. Xiao Y. . (2025). Unlocking biological insights from differentially expressed genes: Concepts, methods, and future perspectives. J. Adv. Res. 76, 135–157. doi: 10.1016/j.jare.2024.12.004, PMID: 39647635 PMC12793742

[B48] Zhang J.-Y. Cun Z. Chen J.-W. (2020). Photosynthetic performance and photosynthesis-related gene expression coordinated in a shade-tolerant species Panax notoginseng under nitrogen regimes. BMC Plant Biol. 20, 273. doi: 10.1186/s12870-020-02434-z, PMID: 32593292 PMC7321538

[B49] Zhang H. Lu L. (2024). Transcription factors involved in plant responses to cadmium-induced oxidative stress. Front. Plant Sci. 15, 2024. doi: 10.3389/fpls.2024.1397289, PMID: 38938636 PMC11209895

[B50] Zhang X. Yang M. Yang H. Pian R. Wang J. Wu A.-M. (2024). The uptake, transfer, and detoxification of cadmium in plants and its exogenous effects. Cells 13, 907. doi: 10.3390/cells13110907, PMID: 38891039 PMC11172145

[B51] Zhang M. Zhang S. (2022). Mitogen-activated protein kinase cascades in plant signaling. J. Integr. Plant Biol. 64, 301–341. doi: 10.1111/jipb.13215, PMID: 34984829

[B52] Zhao B. Erwin A. Xue B. (2018). How many differentially expressed genes: A perspective from the comparison of genotypic and phenotypic distances. Genomics 110, 67–73. doi: 10.1016/j.ygeno.2017.08.007, PMID: 28843784

[B53] Zheng S. Qi J. Fu T. Chen Y. Qiu X. (2023). Novel mechanisms of cadmium tolerance and Cd-induced fungal stress in wheat: Transcriptomic and metagenomic insights. Ecotoxicol. Environ. Saf. 256, 114842. doi: 10.1016/j.ecoenv.2023.114842, PMID: 37027945

