# Supplementary material for: Comparative transcriptome analysis reveals cadmium tolerance mechanisms in two Amaranthus varieties
Source: Front Plant Sci. 2026 Feb 12;17:1724716. doi: 10.3389/fpls.2026.1724716 (PMC12935918; doi:10.3389/fpls.2026.1724716)
Supplement: Supplementary file 1 [file Image1.pdf]

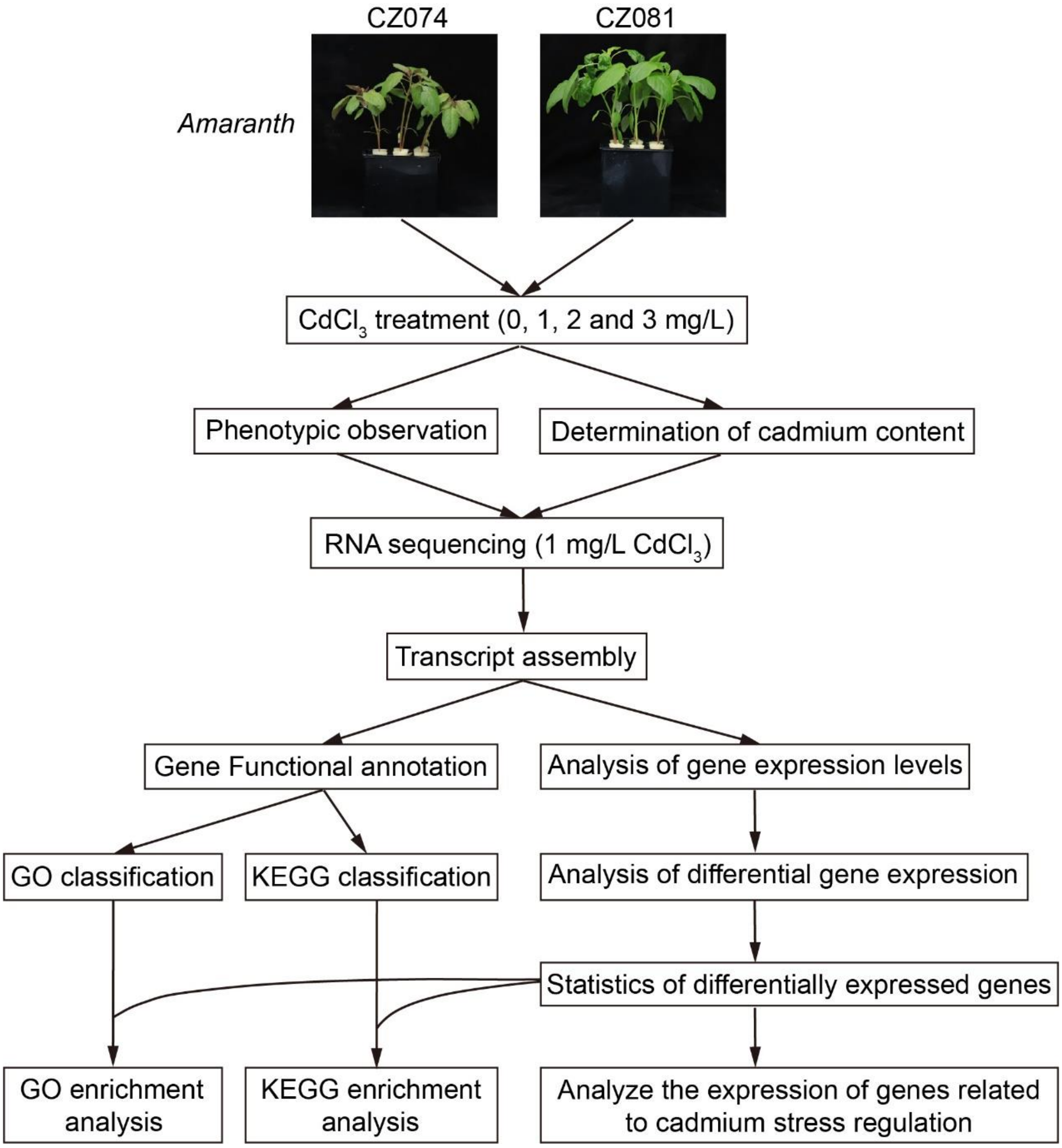

Supplementary Figure 1 Technical workflow of this study.

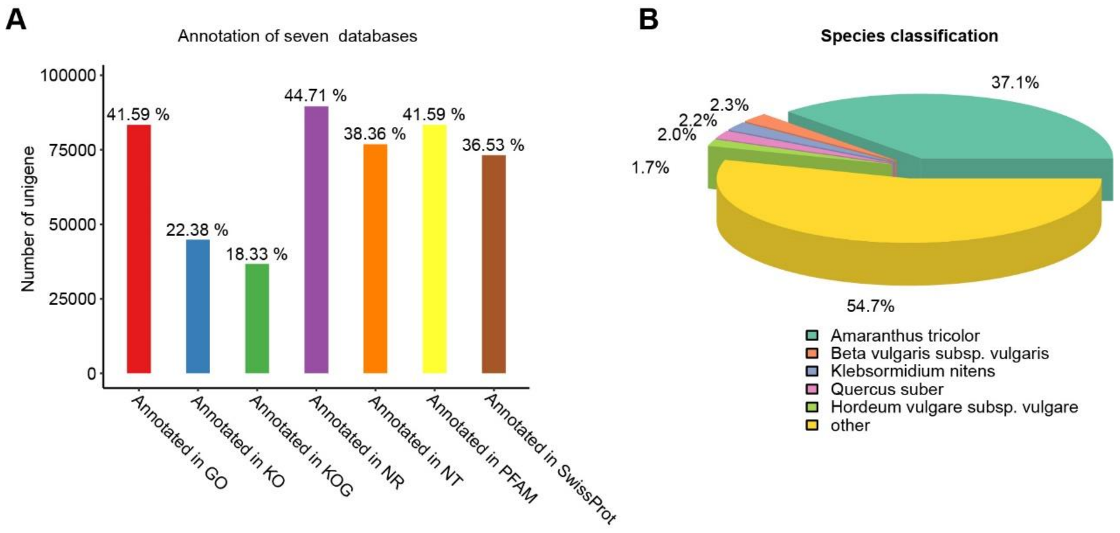

Supplementary Figure 2 Quality and Annotation Assessment of Transcriptome Assembly. **(A)** Gene annotation statistics across seven databases; **(B)** Species distribution of NR database annotation results.

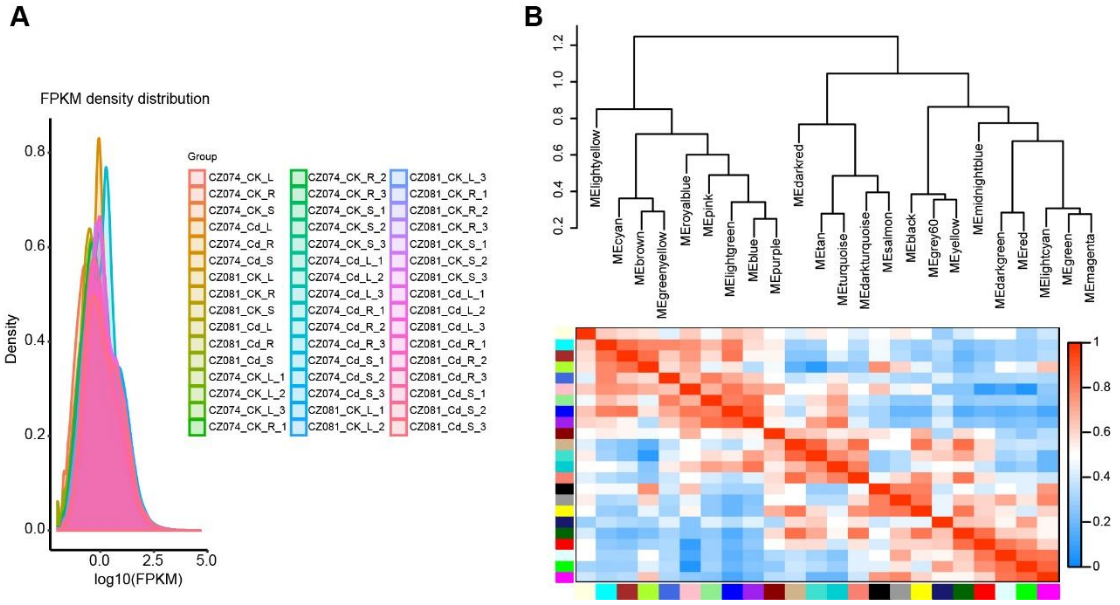

Supplementary Figure 3 Supplementary figures for transcriptome data quality control. **(A)** FPKM density distribution plot for all samples; **(B)** Gene co-expression modules obtained from WGCNA analysis.

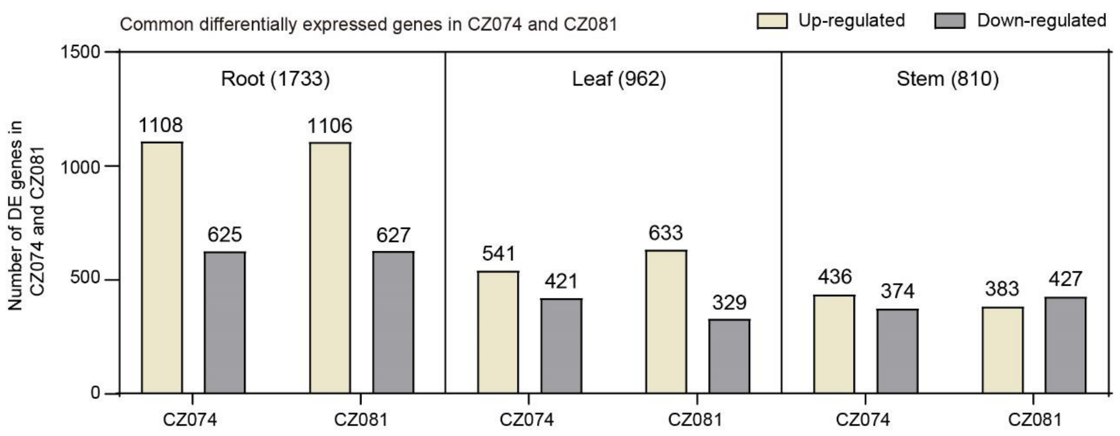

Supplementary Figure 4 Detailed statistics of the numbers of up-regulated and down-regulated common DEGs in roots, stems, and leaves between CZ074 and CZ081 from Figure 4C.

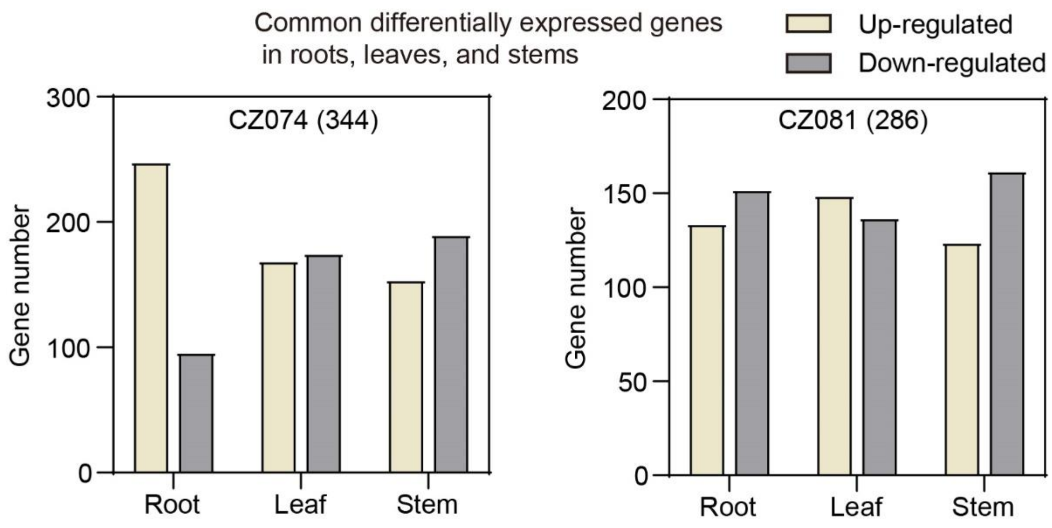

Supplementary Figure 5 Detailed statistics of the numbers of up-regulated and down-regulated common cross-tissue DEGs for CZ074 and CZ081 from Figure 5.

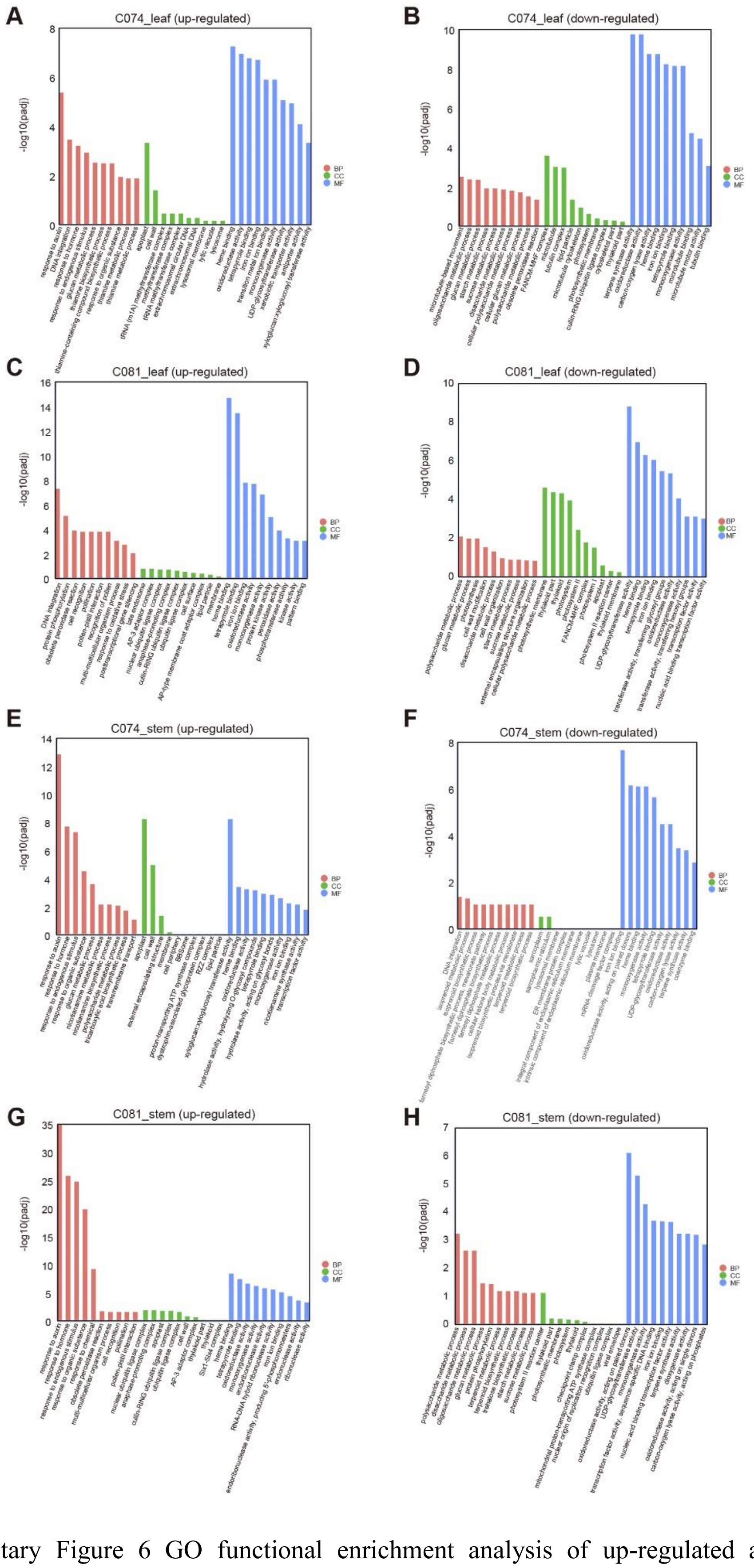

Supplementary Figure 6 GO functional enrichment analysis of up-regulated and down-regulated DEGs in leaf **(A-D)** and stem **(E-H)** tissues of CZ074 and CZ081.

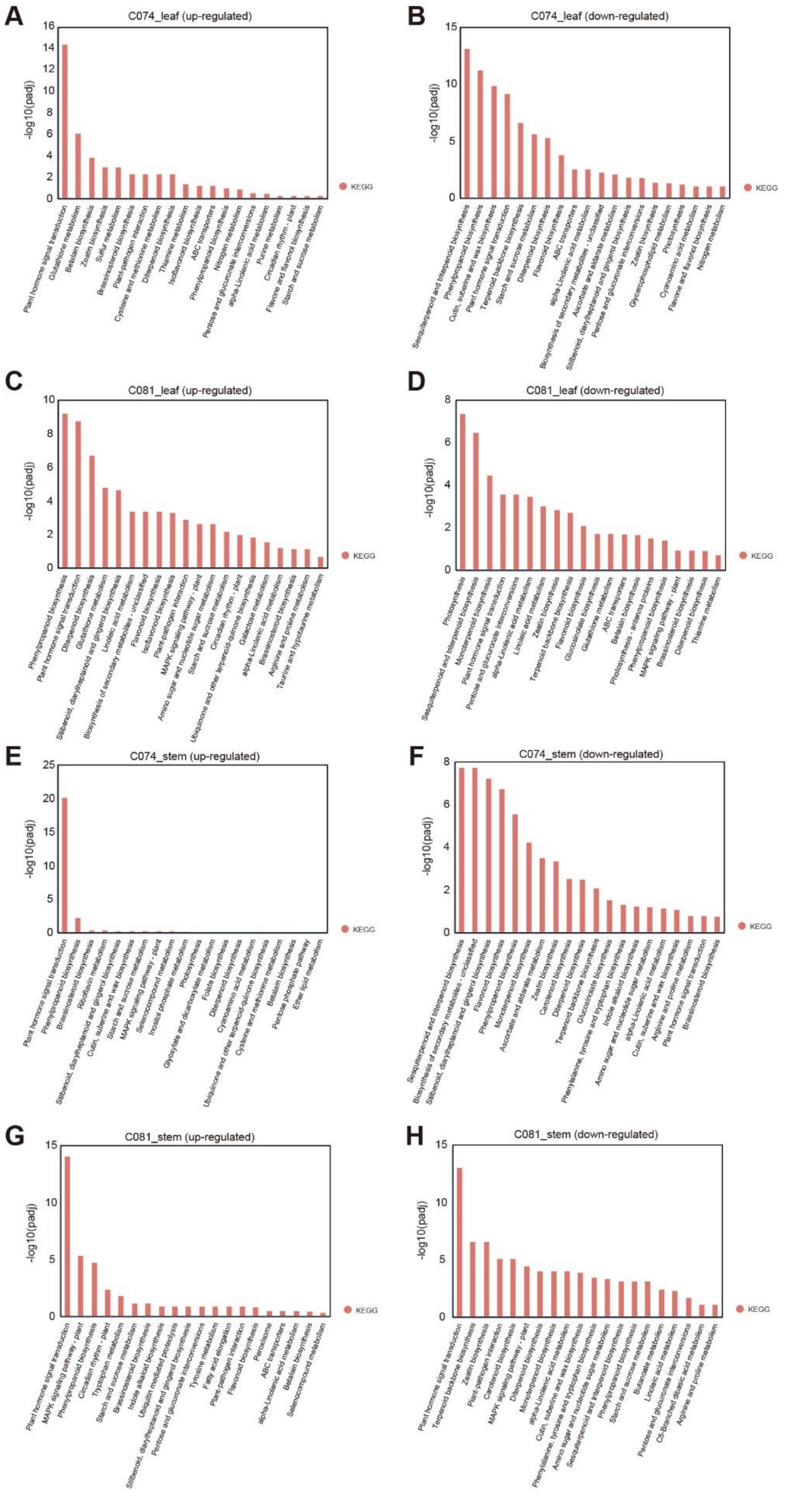

Supplementary Figure 7 KEGG pathway enrichment analysis of DEGs in leaf **(A-D)** and stem **(E-H)** tissues of CZ074 and CZ081.

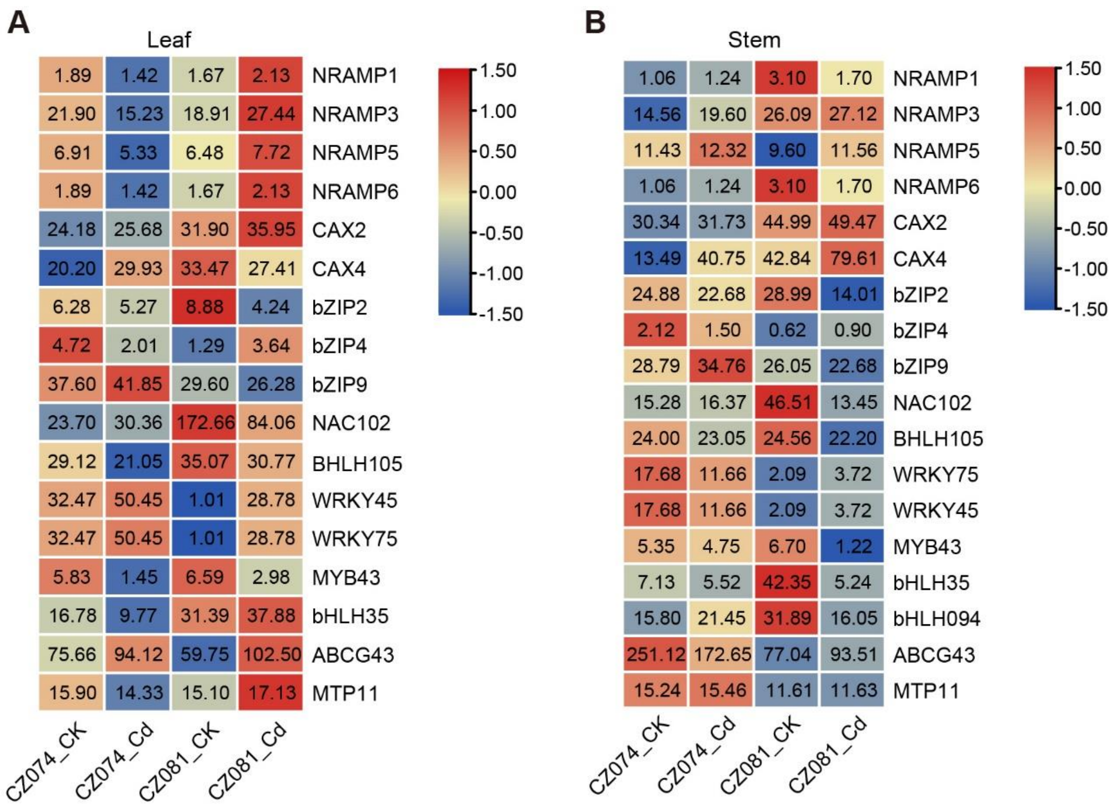

Supplementary Figure 8 Data table showing the expression levels of key cadmium absorption/transport regulators and transcription factors in leaf and stem tissues.
